# Nucleophilic addition and substitution at coordinatively saturated boron by facile 1,2-hydrogen shuttling onto a carbene donor†
†Electronic supplementary information (ESI) available: General experimental details, characterization data for all reported compounds and details of the DFT calculations. CCDC 1563216–1563221. For ESI and crystallographic data in CIF or other electronic format see DOI: 10.1039/c7sc03193a



**DOI:** 10.1039/c7sc03193a

**Published:** 2017-08-04

**Authors:** Dominic Auerhammer, Merle Arrowsmith, Holger Braunschweig, Rian D. Dewhurst, J. Oscar C. Jiménez-Halla, Thomas Kupfer

**Affiliations:** a Institut für Anorganische Chemie , Julius-Maximilians-Universität Würzburg , Am Hubland , 97074 Würzburg , Germany . Email: h.braunschweig@uni-wuerzburg.de; b Institute for Sustainable Chemistry & Catalysis with Boron , Julius-Maximilians-Universität Würzburg , Am Hubland , 97074 Würzburg , Germany; c Departamento de Química , Universidad de Guanajuato , Noria Alta S/N , 36050 Guanajuato , Mexico

## Abstract

The reaction of [(cAAC^Me^)BH_3_] (cAAC^Me^ = 1-(2,6-^i^Pr_2_C_6_H_3_)-3,3,5,5-tetramethylpyrrolidin-2-ylidene) with a range of organolithium compounds led to the exclusive formation of the corresponding (dihydro)organoborates, Li^+^[(cAAC^Me^H)BH_2_R]^–^ (R = sp^3^-, sp^2^-, or sp-hybridised organic substituent), by migration of one boron-bound hydrogen atom to the adjacent carbene carbon of the cAAC ligand. A subsequent deprotonation/salt metathesis reaction with Me_3_SiCl or spontaneous LiH elimination yielded the neutral cAAC-supported mono(organo)boranes, [(cAAC^Me^)BH_2_R]. Similarly the reaction of [(cAAC^Me^)BH_3_] with a neutral donor base L resulted in adduct formation by shuttling one boron-bound hydrogen to the cAAC ligand, to generate [(cAAC^Me^H)BH_2_L], either irreversibly (L = cAAC^Me^) or reversibly (L = pyridine). Variable-temperature NMR data and DFT calculations on [(cAAC^Me^H)BH_2_(cAAC^Me^)] show that the hydrogen on the former carbene carbon atom exchanges rapidly with the boron-bound hydrides.

## Abstract 2

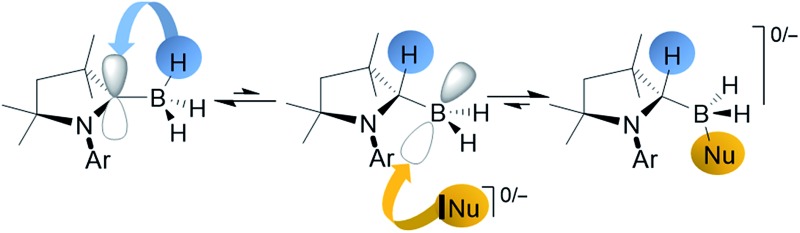
Anionic organic sp^3^-, sp^2^- and sp-nucleophiles as well as neutral Lewis bases were shown to add to a coordinatively saturated cyclic (alkyl)(amino)carbene (cAAC) supported borane thanks to reversible transfer of a hydrogen atom from boron to the adjacent Lewis acidic cAAC carbene carbon atom.

## Introduction

**Fig. 1:**
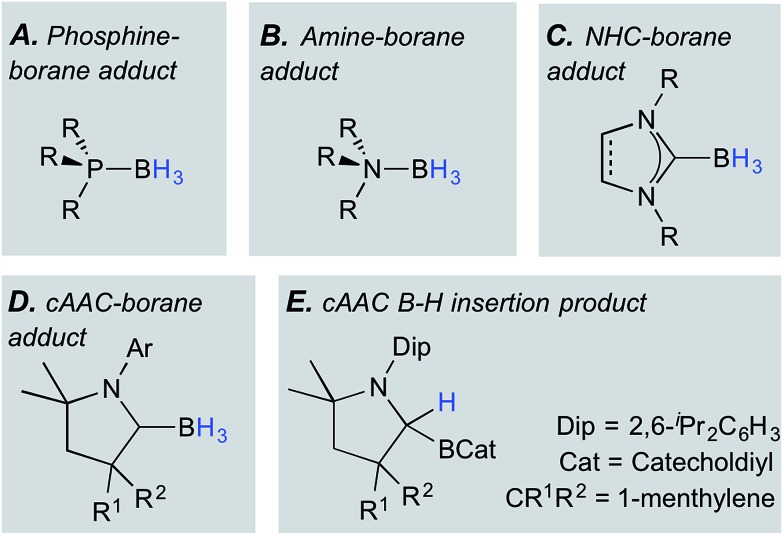
Relevant classes of borane adducts (A–D) and a B–H insertion product (E).

Hydroboranes, particularly BH_3_, are classical Lewis acid reagents used for binding, protecting, or locking the stereochemistry of Lewis basic units in molecules, most commonly applied in the quaternisation of phosphines to form adducts of the form (R_3_P)BH_3_ (Fig. 1A).^1,2^ Similarly, the adduct formation of hydroboranes with amines yields amine–boranes (Fig. 1B), a well-known family of potential hydrogen storage materials.^3^ More recently, a wide range of N-heterocyclic carbenes (NHCs) have been shown to form stable adducts with boranes (Fig. 1C),^4^ a fact that has been impressively exploited by the groups of Fensterbank, Lacôte, Malacria and Curran given their fascinating radical (and other) reactivity patterns.^5^

Cyclic (alkyl)(amino)carbenes (cAACs),^6^ being superficially similar to NHCs, have likewise been used as Lewis bases to bind hydroboranes. The π-electron deficiency at the carbene carbon atom of cAACs, and their consequent ability to accept electrons or other groups at this position, is now well-documented.^7^ Despite this tendency, cAACs have in some cases formed well-behaved adducts (Fig. 1D) with trivalent hydroboranes (*e.g.* BH_3_, BH_2_(CN), and BH(CN)_2_), providing (a) substrates for the unprecedented deprotonation of B–H-containing species,^8^ (b) precursors to Bertrand's archetypal doubly base-stabilised monovalent boron (B^I^) species,^9^ and (c) a precursor to an unusual tetra(B^I^) molecular square.^10^

Early on in this chemistry, the willingness of the cAAC ylidenic carbon atom to accept electron density once bound to a Lewis acidic group became apparent as the reaction of a cAAC with catecholborane led to insertion of the carbene carbon into the B–H bond (Fig. 1E).^7^ Related insertions of cAACs into B–H bonds, and even reversible insertions into B–C bonds, have been reported recently by Marder and Radius.^11^ Similarly, Stephan and Bertrand's reaction of a linear monovalent boron species with H_2_ resulted in formal addition across the B–C_cAAC_ bond, rationalised as an initial hydrogenation of the B(i) unit, followed by 1,2-migration of one hydrogen.^12^ This confirmed Brown's calculations that such a migration is energetically favorable and has a small energy barrier.^13^ Notably also, the groups of Fensterbank, Malacria, Lacôte and Curran reported that their attempt at isolating a novel (cAAC)BH_3_ adduct failed despite solution data showing the formation of the desired adduct and its persistence over 15 h in solution.^14^ It is conceivable that this is due to the non-innocence of the cAAC unit – in particular the willingness of the donor's carbene carbon atom to accept electron density and rehybridise to sp^3^.

In recent work, we have sought to broaden the range of carbene–hydroborane adducts that will undergo deprotonation beyond Bertrand's cAAC adduct of the highly electron-poor borane HB(CN)_2_.^8*a*^ Preliminary NBO calculations led us to consider the adduct (cAAC^Me^)BH_3_ (**1**, cAAC^Me^ = 1-(2,6-^i^Pr_2_C_6_H_3_)-3,3,5,5-tetramethylpyrrolidin-2-ylidene) as a possible candidate given the slight positive charge borne by one of its boron-bound hydrogens.^8*b*^ Our attempts to deprotonate this species with alkyl lithium reagents, however, led not to the expected cAAC-stabilised boryl anion [(cAAC^Me^)BH_2_]^–^, but to a highly unusual nucleophilic alkylation of the coordinatively saturated boron atom and 1,2-hydrogen migration to the cAAC carbene center. This work is presented herein, along with the demonstration of subsequent hydride abstraction allowing a two-step nucleophilic substitution protocol at a sp^3^ boron atom, and reversible 1,2-hydrogen shuttling in the presence of neutral Lewis bases.

## Results and discussion

### Nucleophilic substitution with organolithium bases

**Scheme 1:**
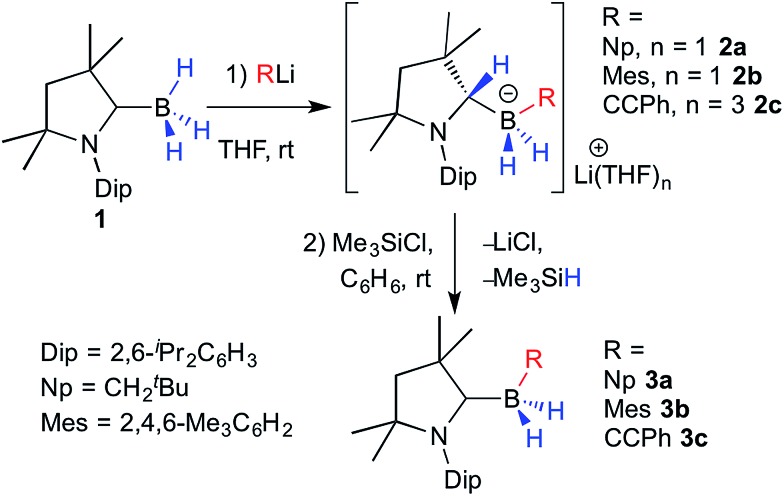
Concomitant organolithiation/hydrogen migration of **1** and subsequent salt elimination/deprotonation to the cAAC-supported (dihydro)organoboranes **3a–c**.

The reaction of **1** with an equimolar amount of NpLi (Np = neopentyl) in THF at room temperature resulted in complete disappearance of the ^11^B NMR quartet of **1** at –33 ppm (^1^ *J*(^11^B–^1^H) = 87 Hz) and the appearance of a new triplet downfield of **1** at –20.4 ppm (^1^ *J*(^11^B–^1^H) = 68 Hz), at first suggesting successful deprotonation. ^1^H NMR data, however, revealed a highly unsymmetrical species, with all cAAC^Me^ protons split into two distinct sets of resonances. A ^1^H{^11^B} NMR spectrum also showed two broad B*H* multiplets centered at 0.25 and 0.02 ppm, respectively, correlating by COSY with two 1H methylene multiplets at 1.07 and 0.92 ppm from the boron-bound neopentyl ligand and a 1H multiplet at 3.45 ppm. Furthermore the ^13^C NMR resonance for the cAAC^Me^ carbene carbon was conspicuously absent from both the ^13^C{^1^H} and HMBC spectra, replaced instead by a broad tertiary carbon resonance at *δ*(^13^C) 69.1 ppm. These NMR data all point to B–H bond activation by the cAAC^Me^ ylidene carbon to yield the lithium (dihydro)neopentylborate species **2a** (Scheme 1). Bertrand and co-workers have previously reported the B–H bond activation of the particularly electron-rich pinacolborane by the π-acidic carbene center of free cAAC ligands.^7^ Similarly, Chiu and co-workers showed that a cAAC adduct of the [Cp*B]^2+^ dication underwent hydride addition at the ylidene carbon upon reaction with [^*n*^Bu_4_N]^+^[BH_4_]^–^.^15^ To our knowledge, however, a 1,2-hydrogen shift from boron to a carbene unit has never been reported.

**Fig. 2:**
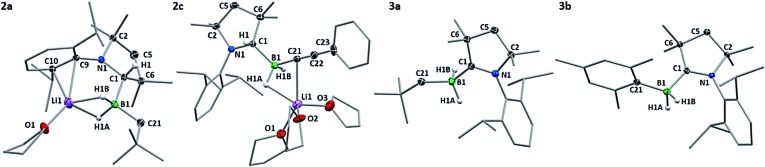
Crystallographically-determined solid-state structures of **2a**, **2c**, **3a** and **3b**. Atomic displacement ellipsoids depicted at 50% probability level and omitted for the ligand periphery. Hydrogen atoms omitted except for those bound to B1 and C1. Selected bond lengths (Å) and angles (°): **2a** N1–C1 1.4863(18), B1–C1 1.643(2), B1–C21 1.635(2), B1–H1A 1.178(18), B1–H1B 1.165(18), B1–Li1 2.284(3), Li1–H1A 1.898(18), Li1–H1B 1.789(18), Li1–C9 2.458(3), Li1–C10 2.467(3), N1–C1–C6 100.47(11), C1–B1–C21 112.94(12); **2c** N1–C1 1.4831(18), B1–C1 1.627(2), B1–C21 1.593(2), B1–H1A 1.180(19), B1–H1B 1.148(19), B1–Li1 2.423(3), C21–C22 1.209(2), Li1–H1A 1.962(18), Li1–C21 2.490(3), N1–C1–C6 101.15(11), C1–B1–C21 112.22(13), B1–C21–C22 177.61(16); **3a** N1–C1 1.3112(17), B1–C1 1.604(2), B1–C21 1.635(2), B1–H1A 1.240(16), B1–H1B 1.116(15), N1–C1–C6 108.50(11), C1–B1–C21 114.55(12); **3b** N1–C1 1.316(2), B1–C1 1.611(3), B1–C21 1.630(3), B1–H1A 1.11(2), B1–H1B 1.10(2), N1–C1–C6 108.74(14), C1–B1–C21 118.28(14).

Compound **2a** crystallised from THF as the mono-THF adduct (Fig. 2). X-ray crystallographic analysis showed the four-coordinate borate center to bear two hydrides (detected in the difference Fourier map and freely refined), a neopentyl residue and the protonated cAAC ligand, which displays sp^3^ hybridisation at C1 (N1–C1 1.4863(18) Å, C6–C1 1.5626(19) Å, N1–C1–C6 100.47(11)°). The lithium counterion, which coordinates to both boron-bound hydrides and a THF ligand, is further supported by π-interaction with the aromatic Dip substituent of the protonated cAAC unit.

**Fig. 3:**
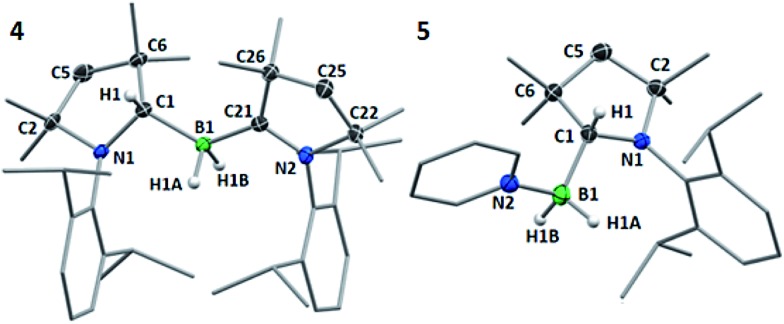
Crystallographically-determined solid-state structures of **4** and **5**. Atomic displacement ellipsoids depicted at the 50% probability level and omitted for the ligand periphery. Hydrogen atoms omitted except for those bound to B1 and C1. Selected bond lengths (Å) and angles (°) for **4**: N1–C1 1.495(2), N2–C21 1.323(2), B1–C1 1.647(2), B1–C21 1.606(3), B1–H1A 1.16(2), B1–H1B 1.14(2), N1–C1–C6 102.00(13), N2–C21–C26 108.00(15), C1–B1–C21 127.87(15); for **5**: N1–C1 1.4770(18), B1–C1 1.628(2), B1–N2 1.615(2), B1–H1A 1.108(17), B1–H1B 1.136(17), N1–C1–C6 101.37(11), C1–B1–N2 109.18(13).

In order to convert the lithium organoborate **2a** to the corresponding neutral borane, one equivalent of Me_3_SiCl was added to a solution of **2a** in C_6_D_6_ (Scheme 1).^16^ After 24 hours at room temperature the ^11^B NMR spectrum of the reaction mixture showed complete consumption of **2a** and a new high-field triplet at –22.2 ppm (^1^ *J*(^11^B–^1^H) = 82 Hz). The ^1^H NMR methine resonance of the protonated cAAC^Me^ ligand had disappeared and ^13^C NMR data showed the diagnostic broad ylidene carbon resonance of a boron-bound neutral cAAC^Me^ ligand at 241.9 ppm, thus enabling identification of the reaction product as the cAAC^Me^-supported (dihydro)neopentylborane **3a** (Scheme 1). This was borne out by the X-ray crystallographic analysis of **3a** (Fig. 3), which clearly shows the now sp^2^-hybridised C1 atom (C1–N1 1.3112(17) Å, ∑(C1) = 359.8(2)°). Whereas organolithiation of neutral boranes usually requires an sp^2^-borane precursor or its adduct with a weakly coordinating Lewis base (SMe_2_, Et_2_O), which is displaced in the process,^17^ the π-acidic cAAC ligand acts in **1** as a reversible hydrogen shuttle, enabling the direct organolithiation and delithiation of an sp^3^-hybridised hydroborane.

In order to test the scope of this reactivity, compound **1** was combined with mesityllithium and lithium phenylacetylide in THF (Scheme 1). In both cases NMR data of the reaction mixture showed the rapid disappearance of the ^11^B NMR quartet of **1** and the appearance of a new *B*H_2_ triplet at –23.0 and –30.4 ppm, respectively (^1^ *J*(^11^B–^1^H) = 79 and 77 Hz, respectively), with ^7^Li NMR spectra displaying broad resonances at –0.13 and –0.34 ppm, respectively. Similarly to **2a**, ^1^H{^11^B} NMR spectra displayed a multiplet at 3.68 and 3.80 ppm for the mesityl (**3b**) and the phenylacetylide derivative (**3c**), respectively, coupling to a broad ^13^C NMR quartet at *ca.* 68 ppm (^1^ *J*(^11^B–^13^C) = 49.7 Hz), attributable to the protonated C1 position of the former cAAC ligand. Another broad ^13^C NMR quartet at 156.5 ppm (^1^ *J*(^11^B–^13^C) = 49.2 Hz) was attributed to the boron-bound mesityl *ipso*-carbon in **2b**, while **2c** displayed a broad ^13^C NMR signal at 111.9 ppm for the boron-bound acetylide carbon.

Single crystals of **2c** obtained from a saturated THF solution confirmed the presence of the two boron-bound hydrides (detected in the difference Fourier map and freely refined), the phenylacetylide ligand and the protonated cAAC^Me^ ligand (Fig. 2). Unlike in **2a**, the lithium counterion is bound to three THF molecules and only one of the boron–borne hydrides, and stabilised by a π interaction with the acetylide C≡C triple bond rather than the aromatic Dip substituent. Neither completely clean NMR spectra nor single crystals of **2b** could be obtained as the compound underwent spontaneous transformation in solution to the corresponding cAAC-supported (dihydro)mesitylborane, **3b** (10% conversion at room temperature over 24 h).‡
‡While this transformation requires formal LiH elimination, the exact mechanism of this reaction remains unclear.


Hydrides of both **2b** and **2c** could be smoothly abstracted to afford the corresponding neutral (dihydro)organoborane cAAC^Me^ adducts, **3b** and **3c**, respectively, using Me_3_SiCl in benzene (Scheme 1). The X-ray crystallographic structure of the mesityl derivative, **3b** (Fig. 3), confirmed that the boron center is coordinated by the neutral cAAC^Me^ ligand, two hydrides and a mesityl ligand. The synthesis of compounds **3a–c** shows that nucleophilic substitution at **1** should be feasible with any sp-, sp^2^- or sp^3^-hybridised organolithium precursor, independently of steric factors. In all three cases, the π-acidic cAAC^Me^ carbene center acts as a temporary repository for one boron-bound hydrogen atom upon organolithiation, transferring that hydrogen back to boron upon delithiation.

**Fig. 4:**
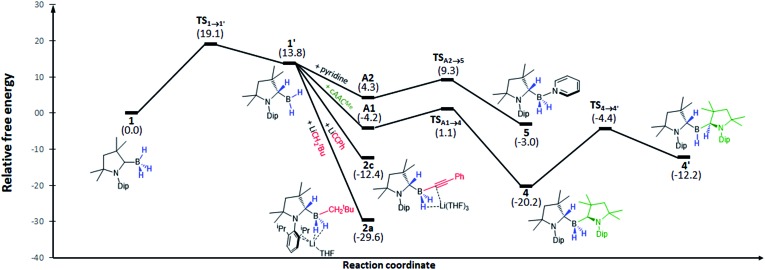
Energy profile of the addition of anionic and neutral bases to **1** and the fluxional behavior of **4** calculated at the SMD(thf):ONIOM(M06-2X/6-311+G(d):PM6) level. Energies shown are in kcal mol^–1^.

In order to understand this hydrogen shuttling mechanism better, DFT calculations were performed at the ONIOM(M06-2X/6-311+G(d):PM6) level using THF as a solvent (see ESI† for further details). Having tested various mechanistic pathways, it became apparent that the initial step is the tautomerisation from the sp^3^-trihydroborane **1** to the sp^2^-(dihydro)organoborane **1′**, by transfer of one hydrogen from boron to the adjacent cAAC carbene carbon atom. This step is very endothermic/endergonic (Δ*H*01 = 14.3 kcal mol^–1^, Δ*G*01 = 13.8 kcal mol^–1^) with an energy barrier of Δ*G*‡1 = 19.1 kcal mol^–1^ (Δ*H*‡1 = 17.4 kcal mol^–1^) and **1′** itself is highly unstable (Fig. 4). In the presence of a strong base, such as LiNp or LiCCPh, however, **1′** undergoes facile addition of R^–^ to yield **2a** and **2c**. The absence of potential transition states connecting **1′** to these products may be explained by the strong polarisation of the Li–C bond, which is easily cleaved, enabling the negatively charged organic fragment to bind directly to the boron atom in **1′**. As a consequence, these are highly exergonic reactions (Δ*G*02 = –26.2 kcal mol^–1^ for LiCCPh, Δ*G*02 = –43.4 kcal mol^–1^ for LiNp, see Fig. 4).

### Addition of neutral bases

Encouraged by the ease of nucleophilic substitution with anionic organic bases, the reactivity of **1** towards neutral donor bases was also investigated. Upon combining equimolar amounts of **1** and the neutral ligand cAAC in C_6_D_6_, ^11^B NMR spectroscopic data showed instant and quantitative conversion to a single new species, compound **4**, presenting a very broad resonance at –18.9 ppm. The ^1^H{^11^B} NMR spectrum displayed a single set of cAAC^Me^ ligand signals integrating in a 2 : 1 ratio with a very broad B*H* resonance centered at 2.15 ppm (full width at half height (fwhh) ≈ 40 Hz) integrating for 3H. The ^13^C{^1^H} NMR spectrum also showed a single set of cAAC^Me^ ligand signals and no evidence of a low-field carbene resonance; instead a very broad quaternary B*C* resonance at 153.4 ppm was observed (fwhh ≈ 170 Hz), corresponding neither to the neutral carbene nor to the C1-protonated ligand. A low-temperature ^1^H NMR experiment at –40 °C in CD_2_Cl_2_ showed splitting of the cAAC^Me^ resonances into two 1 : 1 sets of broadened resonances, and a B*H* resonance centered at 2.40 ppm now integrating for 2H but still no evidence of a protonated cAAC ligand.

**Scheme 2:**
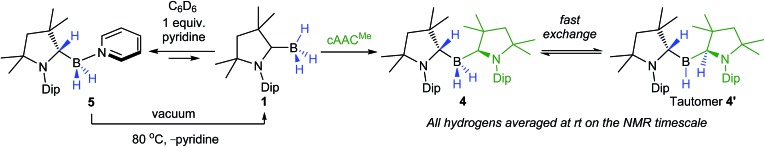
Addition of neutral bases to **1**.

Multiple X-ray diffraction experiments on single crystals of **4** obtained from THF or CH_2_Cl_2_ solutions stored at –30 °C always showed a cAAC^Me^-supported dihydroborane bearing an additional C1-protonated cAAC unit (Fig. 3). While one cAAC residue displays a trigonal planar geometry indicative of sp^2^-hybridisation at the C1 atom (N2–C21 1.323(2) Å, N2–C21–C26 108.00(15)°), the other displays a distorted tetrahedral geometry at C1 indicating sp^3^-hybridisation (N1–C1 1.495(2) Å, N1–C1–C6 102.00(13)°). DFT calculations at the previous level of theory showed that the addition of a cAAC^Me^ molecule to tautomer **1′** to form the pre-adduct **A1** provides significant stabilisation (Δ*G* = –18.0 kcal mol^–1^). The formation of **4** then occurs with a low energy barrier of 5.3 kcal mol^–1^ (Fig. 4). The total reaction energy for the formation of **4** from **1** (Δ*G*0R = –20.3 kcal mol^–1^) is intermediate between that of **2a** (–29.6 kcal mol^–1^) and **2c** (–12.4 kcal mol^–1^). Further calculations were aimed at investigating the intramolecular hydrogen exchange within **4** depicted in Scheme 2. A stable tautomer of **4**, the (diorgano)hydroborane **4′**, resulting from the transfer of a second hydrogen from boron to the carbene carbon of the formerly neutral cAAC ligand, was located 8 kcal mol^–1^ above the energy of **4** (Fig. 4). **4** and **4′** are in fast exchange through a transition state involving the migration of a single hydrogen atom between one of the cAAC ligands and the BH unit, with an energy barrier of 15.8 kcal mol^–1^. This low barrier, which may be easily reached at room temperature, is in agreement with the fluxionality of the system observed by NMR spectroscopy.

In pyridine solution compound **1** similarly underwent full conversion to the corresponding pyridine-stabilised dihydroborane bearing the protonated cAAC^Me^ ligand, compound **5**, as evidenced by the appearance of a new broad ^11^B NMR resonance at –4.4 ppm. An X-ray crystallographic experiment performed on single crystals of **5** obtained at –30 °C confirmed the presence of the boron-bound pyridine molecule, the two boron-bound hydrides and the sp^3^-hybridised C1 carbon atom (N1–C1 1.4770(18) Å, N1–C1–C6 101.37(11)°) of the cAACH unit (Fig. 4).

Isolated crystals of **5** always showed *ca.* 10% of free pyridine and **1** in their NMR spectra when dissolved in C_6_D_6_ at room temperature. In order to investigate whether this might be the result of decomposition or of reversible pyridine addition to **1**, a variable-temperature NMR experiment was performed on a 0.11 mM solution of **5** in C_6_D_6_ with temperatures ranging from 15 to 70 °C (Fig. S31–S32†). This indeed showed a reversible process, with a 3 : 7 ratio of free pyridine (or **1**) to **5** at 70 °C. For the forward reaction depicted, a van't Hoff analysis provided a reaction enthalpy of Δ*H* ≈ –12 kcal mol^–1^ and a very negative reaction entropy of Δ*S* ≈ –40.3 cal mol^–1^ K^–1^, reflecting the reduced molecularity of product *versus* reactants (Fig. S33†). At room temperature the Gibbs free energy for the forward reaction is negligibly negative (Δ*G*(298 K) ≈ –9 cal mol^–1^), *i.e.* only slightly in favor of **5**, as observed by NMR spectroscopy. DFT modelling of the reaction leads to the same reaction mechanism as for the formation of **4**: first, the addition of pyridine to **1′** lowers the energy by –9.5 kcal mol^–1^ (pre-adduct **A2**), then compound **5** is formed *via* a transition state with an associated energy barrier of only 5.0 kcal mol^–1^ (Fig. 4). The total reaction energy is only –3.0 kcal mol^–1^ (which corresponds to Δ*G*(298.15 K) = –13.8 kcal mol^–1^, close to the experimental value above). The energy barrier of the reverse reaction (going from **5** to the transition state **TS_1→1′_**) is 22.0 kcal mol^–1^, which can be easily achieved through heating. Such reversibility is not possible for the other bases, which are stronger σ-donors, as can be seen upon comparison of their respective inverse energy barriers (31.5 kcal mol^–1^ for LiCCPh, 39.3 kcal mol^–1^ for cAAC^Me^ and 48.7 kcal mol^–1^ for LiNp).

Interestingly, the reaction was reversible even in the solid state, as heating compound **5** to 80 °C under vacuum resulted in quantitative recovery of compound **1**, the pyridine having been removed *in vacuo* (Scheme 2). In contrast, compound **4** could be heated to 200 °C *in vacuo* without any evidence of decomposition.

## Conclusions

These results have uncovered the facile nucleophilic addition to the coordinatively saturated sp^3^ boron atom in (cAAC^Me^)BH_3_ with a range of anionic sp, sp^2^ and sp^3^ organic nucleophiles, as well as neutral Lewis bases. Computational analyses show that the sp^2^-hybridised tautomer of (cAAC)BH_3_, in which one hydrogen has migrated from boron to the adjacent C_cAAC_ atom, is a common intermediate to all these nucleophilic addition reactions. While the addition of relatively weakly Lewis basic pyridine occurs reversibly, the addition of a strongly Lewis basic cAAC^Me^ ligand forms a stable product, which undergoes fluxional swapping of all three hydrogen atoms between the boron atom and two cAAC units *via* a (diorgano)hydroborane intermediate, in which both cAAC moieties are protonated.

The discovery of a facile and reversible B ↔ C 1,2-hydrogen migration process in cAAC–borane adducts underlines the now well-established non-innocence of the carbene carbon of cAAC donors.^6^ More importantly, however, these results provide essential caveats for researchers seeking to explore the combination of cAACs (and other π-acidic carbenes) with hydroboranes: (a) that boron-bound hydrogen atom(s) may be shuttling between the boron and C_cAAC_ atoms in the presence of bases (or even in their absence), which has implications for the spectroscopic identification of reaction products, and (b) that even relatively weak bases will bind to the boron atom in these adducts, despite its apparent coordinative saturation. The reversible hydrogen shuttling presented in this work shows parallels with the burgeoning concept of metal–ligand cooperativity, as reviewed recently by Milstein,^18^ and suggests the future use of cAAC ligands in metal- or element-ligand cooperative catalysis.
