# Supplementary material for: Nucleophilic addition and substitution at coordinatively saturated boron by facile 1,2-hydrogen shuttling onto a carbene donor
Source: Chem Sci. 2017 Aug 4;8(10):7066–71. doi: 10.1039/c7sc03193a (PMC5637459; doi:10.1039/c7sc03193a)
Supplement: Supplementary file 1 [file SC-008-C7SC03193A-s001.pdf]

Electronic Supplementary Material (ESI) for Chemical Science.
This journal is © The Royal Society of Chemistry 2017

Supporting information for:

Nucleophilic Addition and Substitution at Coordinatively Saturated Boron by Facile 1,2-Hydrogen Shuttling onto a Carbene Donor

Dominic Auerhammer,[a,b] Merle Arrowsmith,[a,b] Holger Braunschweig,[a,b,]* Rian D. Dewhurst,[a,b] J. Oscar C. Jiménez-Halla,[a,c] Thomas Kupfer[a,b]

[a] *Institut für Anorganische Chemie, Julius-Maximilians-Universität Würzburg, Am Hubland, 97074 Würzburg (Germany).*

[b] *Institute for Sustainable Chemistry & Catalysis with Boron, Julius-Maximilians-Universität Würzburg, Am Hubland, 97074 Würzburg (Germany).*

[c] *Departamento de Química, Universidad de Guanajuato, Noria Alta S/N, 36050 Guanajuato (México).*

Table of contents

Methods and Materials....2
Synthesis and Characterization....2
NMR Spectra....7
Low temperature NMR study of **4**....16
Variable-Temperature NMR Study of **5** in C_6D_6 (0.11 M)....17
Crystallographic Details....18
Computational Details....20
References....40

Methods and Materials

All manipulations were performed either under an atmosphere of dry argon or in vacuo using standard Schlenk line or glovebox techniques. Deuterated solvents were dried over molecular sieves and degassed by three freeze-pump-thaw cycles prior to use. All other solvents were distilled and degassed from appropriate drying agents. Solvents (both deuterated and non-deuterated) were stored under argon over activated 4 Å molecular sieves. NMR spectra were acquired on a Bruker Avance 500 NMR spectrometer (^{1}H: 500.1 MHz, ^{7}Li{^{1}H}: 197.4 MHz, ^{11}B: 160.5 MHz, ^{13}C{^{1}H}: 125.8 MHz or on a Bruker Avance 400 NMR spectrometer (^{1}H: 400.1 MHz, ^{11}B: 128.4 MHz, ^{13}C{^{1}H}: 100.6 MHz. Chemical shifts (δ) are given in ppm and internally referenced to the carbon nuclei (^{13}C{^{1}H}) or residual protons (^{1}H) of the solvent. ^{7}Li{^{1}H} and ^{11}B{^{1}H} NMR spectra were referenced to external standards LiCl and [$BF_3 \cdot OEt_2$], respectively. Unless stated otherwise all NMR spectra of isolated compounds were recorded at 298 K.

Me_3SiCl and pyridine were degassed using freeze-pump-thaw cycles and stored in the glovebox over molecular sieves. The free cAAC^{Me} ligand (1-(2,6-^{i}Pr$_2$C$_6$H$_3$)-3,3,5,5-tetramethylpyrrolidin-2-ylidene),[1] NpLi (Np = CH$_2$^{t}Bu),[2] MesLi(OEt$_2$) (Mes = 2,4,6-Me$_3$C$_6$H$_2$)[3] and PhC≡CLi(OEt$_2$)[4] were synthesized following literature procedures.

Synthesis and Characterization

[(cAAC^{Me})BH$_3$] (1)

BH$_3$(SMe$_2$) (263 mg, 3.50 mmol) was dissolved in hexanes (2.5 mL) and added to a solution of cAAC (1.00 g, 3.50 mmol) in hexanes (2.5 mL). The reaction mixture was stirred at rt for 4 h then filtered. The resulting solid was washed with hexanes (1 mL) and dried in vacuo yielding **1** as a colorless crystalline solid (1.02 g, 3.41 mmol, 97%). ^{1}H{^{11}B} NMR (400 MHz, C$_6$D$_6$): δ = 7.44–7.37 (m, 1H, Ar*H*), 7.30–7.23 (m, 2H, Ar*H*), 2.70 (sept, ^3J = 6.7 Hz, 2H, CH_{iPr}), 2.06 (s, 2H, CH_2), 1.50 (s, 6H, CH_3), 1.34–1.27 (overlapping s, 6H, CH_3, and d, ^3J = 6.9 Hz, 6H, C$H_{3\text{-iPr}}$), 1.21 (d, ^3J = 6.7 Hz, 6H, C$H_{3\text{-iPr}}$), 0.71 (s, 3H, BH_3), ppm. ^{13}C{^{1}H} NMR (100.6 MHz, C$_6$D$_6$): δ = 241.9 (br, C$_{\mathrm{carbene}}$), 145.8, 134.0 (C^q), 129.3, 124.8 (CH$_{\mathrm{Ar}}$), 76.8, 52.4 (C^q), 51.0 (CH$_2$), 30.3 (CH), 29.6, 29.0, 25.8, 23.4 (CH$_3$). ^{11}B NMR (128.4 MHz, C$_6$D$_6$): δ = –32.3 (q, $^1J_{\mathrm{BH}}$ = 87.0 Hz) ppm. Positive-ion ASAP−MS (*m/z*) calculated for [C$_{20}$H$_{34}$BN] = 299.2779; found: 299.2775.

[(cAAC^{Me}-H)BH$_2$Np•Li(thf)] (2a)

Compound **1** (200 mg, 668 μmol) was dissolved in THF (1.0 mL). A solution of neopentyllithium (55.0 mg, 704 μmol) in THF (0.5 mL) was added dropwise over a period of 2 min. After stirring at room temperature for 22 h, all volatiles were removed, leaving a pale yellow solid, which was washed with cold pentane (2 x 0.5 mL). Recrystallization from THF (0.5 mL) provided compound **2a** as colorless crystals (264 mg, 587 μmol, 88%). ^{1}H{^{11}B} NMR (500 MHz, C$_6$D$_6$): δ = 7.00–6.97 (dd, ^3J = 7.6 Hz, ^4J = 1.5 Hz, 1H, Ar*H*), 6.91–6.88 (dd, ^3J = 7.6 Hz, ^4J = 1.5 Hz, 1H, Ar*H*), 6.82 (t, ^3J = 7.6 Hz, 1H, Ar*H*), 4.30 (sept, ^3J = 6.8

Hz, 1H, CH_{iPr}), 3.70 (sept, ^3J = 6.8 Hz, 1H, CH_{iPr}), 3.45 (dd, ^3J = 2.5, 6.5 Hz, 1H, BCH), 3.06 (m, 4H, THF-H), 1.98 (d, 1H, CH_2), 1.95 (d, 1H, CH_2), 1.60 (s, 6H, CH_3), 1.51 (d, ^3J = 6.8 Hz, 3H, C$H_{3\text{-iPr}}$), 1.37 (s, 3H, CH_3), 1.29 (d, ^3J = 6.8 Hz, 3H, C$H_{3\text{-iPr}}$), 1.25 (s, 3H, CH_3), 1.23–1.18 (overlapping d, 3H, C$H_{3\text{-iPr}}$, d, 3H, C$H_{3\text{-iPr}}$, and s, 9H, C(CH_3)$_3$), 1.12 (m, 2H, THF-H), 1.06 (ddd, ^2J = 13.6 Hz, ^4J = 7.7, 3.9 Hz, 1H, BCH_2), 0.92 (ddd, ^2J = 13.6 Hz, ^4J = 6.3, 1.7 Hz, 1H, BCH_2), 0.25 (br m, 1H, BH), 0.02 (br m, 1H, BH) ppm. ^{13}C{^{1}H} NMR (125.8 MHz, C_6D_6): δ = 156.5, 151.7, 144.2 (C^q), 126.3, 124.9, 124.5 (CH$_{Ar}$), 69.1 (br m, identified by HSQC, BCH), 68.2 (THF), 62.2, 62.2 (CH$_2$), 42.0 (C^q), 35.2 (br m, identified by HSQC, BCH$_2$), 32.9, 31.7, 31.7, 29.6 (CH$_3$), 29.0 (CH), 28.0 (CH$_3$), 27.2 (CH), 27.0, 26.7, 26.2 (CH$_3$), 25.1 (THF), 24.9, 24.4 (CH$_3$). ^{11}B NMR (160.5 MHz, C_6D_6): δ = –20.4 (t, $^1J_{BH}$ = 68.1 Hz) ppm. ^{7}Li{^{1}H} NMR (197.4 MHz, C_6D_6): δ = –2.54 ppm. Negative-ion ASAP-MS (*m/z*) calculated for $[C_{25}H_{45}B_1N_1]^-$ = 370.3640; found: 370.3638.

[(cAAC^{Me})BH$_2$Np] (3a)

The lithium borate **2a** (221 mg, 492 µmol) was dissolved in C_6D_6 (1.0 mL) prior to dropwise addition of Me$_3$SiCl (59.0 mg, 543 µmol). The reaction mixture was stirred at rt for 24 h, filtered and the volatiles removed in vacuo. The resulting pale yellow solid was recrystallized from pentane (0.5 mL) to yield **3a** as a colorless crystalline solid (167 mg, 452 µmol, 92%). ^{1}H{^{11}B} NMR (500 MHz, C_6D_6): δ = 7.11 (dd, ^3J = 6.9, 8.3 Hz, 1H, ArH), 7.04 (m, 2H, ArH), 2.70 (sept, ^3J = 6.7 Hz, 2H, CH_{iPr}), 1.67 (t, ^4J = 6.3 Hz, 2H, BH_2), 1.47 (s, 2H, CH_2), 1.44 (s, 6H, CH_3), 1.40 (d, ^3J = 6.8 Hz, 6H, C$H_{3\text{-iPr}}$), 1.32 (s, 9H, CH_3), 1.16 (d, ^3J = 6.8 Hz, 6H, C$H_{3\text{-iPr}}$), 1.01–0.95 (m, 2H, CH_2), 1.98 (t, ^4J = 6.3 Hz, 2H, BCH_2), 0.84 (s, 6H, CH_3) ppm. ^{13}C{^{1}H} NMR (125.8 MHz, C_6D_6): δ = 238.71 (C$_{carbene}$, detected by HMBC), 145.4, 134.2 (C^q), 129.1, 124.4 (CH$_{Ar}$), 74.9, 52.5 (C^q), 52.0 (CH$_2$), 34.3 (br m, BCH$_2$), 32.7 (CH$_{3\text{-tBu}}$), 32.0 (CH$_3$), 29.4 (CH), 29.0, 28.6, 26.1, 23.3 (CH$_3$). ^{11}B NMR (160.5 MHz, C_6D_6): δ = –22.2 (t, $^1J_{BH}$ = 82.2 Hz) ppm. Positive-ion ASAP-MS (*m/z*) calculated for $[C_{25}H_{43}B_1N_1]$ = 368.3483; found: 368.3475.

[(cAAC^{Me}-H)BH$_2$Mes•Li(thf)] (2b)

Compound **1** (100 mg, 334 µmol) was dissolved in THF (2.0 mL). A solution of mesityllithium diethylether complex (67.0 mg, 335 µmol) in THF (1.0 mL) was added dropwise over a period of 2 min. However, **2b** could not be cleanly isolated as it spontaneously decomposed to compound **3b** in solution at room temperature. NMR and MS data of **2b** were therefore measured *in situ* in a reaction carried out in d_8-THF. The number of thf ligands was deduced from the negative ion ASAP-MS results. ^{1}H{^{11}B} NMR (500 MHz, d_8-THF): δ = 6.96–6.94 (m, 1H, ArH), 6.89–6.86 (m, 2H, ArH), 6.39 (s, 2H, Mes-H), 4.36 (sept, ^3J = 6.8 Hz, 1H, CH_{iPr}), 3.68 (br t, ^3J = 5.2 Hz, 1H, BCH), 3.61 (sept, ^3J = 6.8 Hz, 1H, CH_{iPr}), 2.32 (s, 6H, Mes-CH_3), 2.05 (s, 3H, Mes-CH_3), 1.57 (d, ^2J = 11.8 Hz, 1H, CH_2), 1.54 (d, ^2J = 11.8 Hz, 1H, CH_2), 1.37 (d, ^3J = 6.8 Hz, 3H, C$H_{3\text{-iPr}}$), 1.34–1.40 (br, BH), 1.27 (d, ^3J = 6.8 Hz, 3H, C$H_{3\text{-iPr}}$), 1.16–1.22 (br, BH), 1.19 (d, ^3J = 6.8 Hz, 3H, C$H_{3\text{-iPr}}$), 1.16 (d, ^3J = 6.8 Hz, 3H, C$H_{3\text{-iPr}}$), 1.07 (s, 3H, CH_3), 1.00 (s, 3H, CH_3), 0.98 (s, 3H, CH_3), 0.56 (s, 3H, CH_3) ppm. ^{13}C{^{1}H} NMR (125.8 MHz, d_8-THF): δ = 156.5, 153.8, 153.7, 144.7, 143.2 (C^q), 126.9, 124.1, 123.2, 123.1 (CH$_{Ar}$), 68.7 (br, BCH), 62.6 (C^q), 60.8 (CH$_2$), 42.4 (C^q), 30.8, 29.6 (CH$_3$), 29.1 (CH), 28.4, 27.7 (CH$_3$), 27.6 (CH), 27.2, 26.4, 26.2, 25.2, 25.0, 21.0 (CH$_3$). ^{11}B

NMR (160.5 MHz, d_8-THF): δ = –23.0 (t, $^1J_{BH}$ = 78.8 Hz) ppm. ^{7}Li{^{1}H} NMR (197.4 MHz, d_8-THF): δ = –0.13 ppm. Negative-ion ASAP-MS (*m/z*) calculated for $[C_{29}H_{45}B_1N_1]^-$ = 418.3640; found: 418.3651; calculated for $[C_{66}H_{104}B_2LiN_2O_2]^-$ (corresponding to two molecules of **2b** minus LiH, minus H^+) = 985.8438; found: 985.8459.

[(cAAC^{Me})BH$_2$(Mes)] (3b)

The lithium borate **2b** was synthesized *in situ* in THF as described above. The reaction mixture was stirred at rt for 24 h prior to removal of volatiles in vacuo. The resulting crude solid was suspended in benzene (0.6 mL) and Me_3SiCl (36.4 mg, 335 µmol) added dropwise. The reaction mixture was then stirred at rt for 1 d, filtered and crystallized by slow evaporation of the filtrate to yield **3b** as a colorless crystalline solid (117 mg, 281 µmol, 84%). ^{1}H{^{11}B} NMR (500 MHz, C_6D_6): δ = 7.20–7.17 (m, 1H, Ar*H*), 7.12–7.09 (m, 2H, Ar*H*), 7.07 (s, 2H, Mes*H*), 2.76 (sept, ^3J = 6.6 Hz, 2H, CH_{iPr}), 2.65 (s, 6H, Mes-CH_3), 2.53 (s, 2H, BH_2), 2.38 (s, 6H, Mes-CH_3), 1.54 (d, ^3J = 6.6 Hz, 6H, C$H_{3\text{-}iPr}$), 1.40 (s, 2H, CH_2), 1.20 (s, 6H, CH_3), 1.18 (d, ^3J = 6.6 Hz, 6H, C$H_{3\text{-}iPr}$), 0.87 (s, 6H, CH_3), ppm. ^{13}C{^{1}H} NMR (125.8 MHz, C_6D_6): δ = 242.6 ($C_{carbene}$, identified by HMBC), 146.5 (br, B*C*), identified by HMBC), 145.5, 142.9, 133.8, 133.2 (C^q), 129.1, 128.5, 125.4 (CH_{Ar}), 75.3, 53.3 (C^q), 52.6 (CH_2), 29.3 (CH), 29.2, 28.3, 27.4, 25.9, 24.8, 21.4 (CH_3). ^{11}B NMR (160.5 MHz, C_6D_6): δ = –22.9 (t, $^1J_{BH}$ = 83.2 Hz) ppm. LIFDI-MS (*m/z*) calculated for $[C_{29}H_{43}B_1N_1]$ = 416.3483; found: 416.3477.

[(cAAC^{Me}-H)BH$_2$(CCPh)•Li(thf)$_3$] (2c)

Compound **1** (100 mg, 334 µmol) was dissolved in THF (2.0 mL). A solution of lithium phenylacetylide diethylether complex (60.8 mg, 334 µmol) in THF (1.0 mL) was added dropwise over a period of 2 min. After stirring at room temperature for 3 d, all volatiles were removed, leaving a pale yellow solid, which was washed with cold pentane (2 x 0.5 mL). Recrystallization from THF (1.0 mL) provided compound **2c** as colorless crystals (185 mg, 297 µmol, 89%). ^{1}H{^{11}B} NMR (500 MHz, C_6D_6): δ = 7.37 (dm, ^3J = 8.5 Hz, 2H, Ph*H*), 7.25 (dd, ^3J = 7.0 Hz, ^4J = 2.3 Hz, 1H, Ar*H*), 7.19 (dd, ^3J = 7.6 Hz, ^4J = 2.3 Hz, 1H, Ar*H*), 7.16 (m, 1H, Ar*H*), 7.04 (tt, ^3J = 7.4 Hz, ^4J = 1.3 Hz, 2H, Ph*H*), 6.95 (tt, ^3J = 7.4 Hz, ^4J = 1.3 Hz, 1H, Ph*H*), 4.56 (sept, ^3J = 6.8 Hz, 1H, CH_{iPr}), 4.00 (sept, ^3J = 6.8 Hz, 1H, CH_{iPr}), 3.80 (dd, ^3J = 2.8, 6.5 Hz, 1H, BC*H*), 3.43 (m, 12H, THF-*H*), 2.20 (d, 1H, CH_2), 2.13 (d, 1H, CH_2), 1.79 (s, 3H, CH_3), 1.77 (s, 3H, CH_3), 1.56 (d, ^3J = 6.8 Hz, 3H, C$H_{3\text{-}iPr}$), 1.51 (d, ^3J = 6.8 Hz, 3H, C$H_{3\text{-}iPr}$), 1.48 (s, 3H, CH_3), 1.45 (d, ^3J = 6.8 Hz, 3H, C$H_{3\text{-}iPr}$), 1.42 – 1.38 (overlapping d, 3H, C$H_{3\text{-}iPr}$, and s, 3H, CH_3), 1.29 (m, 12H, THF-*H*), 1.05 (dd, ^2J = 11.6 Hz, ^3J = 6.5 Hz, 1H, B*H*), 0.85 (dd, ^2J = 11.6 Hz, ^3J = 2.8 Hz, 1H, B*H*) ppm. ^{13}C{^{1}H} NMR (125.8 MHz, C_6D_6): δ = 153.3, 153.0, 143.4 (C^q), 131.2, 126.3, 125.2, 123.8 (CH_{Ar}), 111.9 (br, B*C*≡C), 99.3 (BC≡*C*), 68.3 (br, B*C*H), 68.3 (THF), 61.9 (C^q), 60.9 (CH_2), 41.2 (C^q), 31.7, 31.0 (CH_3), 29.7 (CH), 29.0, 28.1 (CH_3), 27.4 (CH), 26.9, 25.8 (CH_3), 25.5 (THF), 24.9 (CH_3). ^{11}B NMR (160.5 MHz, C_6D_6): δ = –30.4 (t, $^1J_{BH}$ = 77.1 Hz) ppm. ^{7}Li{^{1}H} NMR (197.4 MHz, C_6D_6): δ = –0.34 ppm. Negative-ion ASAP-MS (*m/z*) calculated for $[C_{28}H_{39}B_1N_1]^-$ = 400.3170; found: 400.3168.

[(cAAC^{Me})BH$_2$(CCPh)] (3c)

The lithium borate **2c** (80.0 mg, 128 µmol) was dissolved in C_6D_6 (0.6 mL) prior to dropwise addition of Me_3SiCl (14.5 mg, 133 µmol). The reaction mixture was stirred at rt for 1 h, filtered and the volatiles removed in vacuo. The resulting pale yellow solid was recrystallized from pentane (0.2 mL) to yield **3c** as a colorless crystalline solid (37.1 mg, 92.9 µmol, 73%). $^1H\{^{11}B\}$ NMR (500 MHz, C_6D_6): δ = 7.35 (dd, ^3J = 6.9, 8.5 Hz, 1H, Ar*H*), 7.27 (m, 2H, Ar*H*), 7.17–7.13 (m, 2H, Ph*H*), 7.12–7.08 (m, 2H, Ph*H*), 7.03 (tt, ^3J = 7.3, ^4J = 1.4, 1H, Ph*H*), 2.75 (sept, ^3J = 6.7 Hz, 2H, CH_{iPr}), 2.10 (s, 2H, CH_2), 1.72 (s, 6H, CH_3), 1.70 (s, 2H, BH_2), 1.34 (s, 6H, CH_3), 1.30 (d, ^3J = 6.7 Hz, 6H, $CH_{3\text{-iPr}}$), 1.27 (d, ^3J = 6.7 Hz, 6H, $CH_{3\text{-iPr}}$) ppm. $^{13}C\{^1H\}$ NMR (125.8 MHz, C_6D_6): δ = 236.40 ($C_{carbene}$), 145.87, 133.95 (C^q), 131.41, 129.84, 129.05, 128.50, 128.14, 125.61, 125.05 (CH_{Ar}), 107.21, 97.0, 77.6, 53.4 (C^q), 51.6 (CH_2), 29.9 (CH), 29.5, 28.8, 25.9, 23.5 (CH_3). ^{11}B NMR (160.5 MHz, C_6D_6): δ = –29.6 (t, $^1J_{BH}$ = 87.4 Hz) ppm. Positive-ion ASAP-MS (*m/z*) calculated for $[C_{28}H_{39}B_1N_1+H]^+$ = 400.3170; found: 400.3162.

[(cAAC^{Me}-H)BH$_2$(cAAC)] (4)

Compound **1** (100 mg, 334 µmol) und cAAC (95.4 mg, 334 µmol) were dissolved in CD_2Cl_2 (0.6 mL) and stirred at room temperature for 24 h. The solution was cooled to –30 °C to obtain colorless crystals of **4** (184 mg, 314 µmol, 94%). $^1H\{^{11}B\}$ NMR (500 MHz, CD_2Cl_2, 298 K): δ = 7.05–7.01 (m, 2H, Ar*H*), 6.99–6.96 (m, 4H, Ar*H*), 2.99 (sept, ^3J = 6.7 Hz, 4H, CH_{iPr}), 2.40–1.92 (br, 3H, BH_3), 1.79 (s, 4H, CH_2), 1.40 (s, 12H, CH_3), 1.14 (d, ^3J = 6.7 Hz, 12H, $CH_{3\text{-iPr}}$), 1.05 (s, 12H, CH_3), 0.78 (broad s, 12H, $CH_{3\text{-iPr}}$) ppm. $^{13}C\{^1H\}$ NMR (125.8 MHz, CD_2Cl_2): δ = 153.4 (br, B*C*), 149.2, 139.1, 128.2 (C^q), 126.5, 124.4 (CH_{Ar}), 68.3 (C^q), 57.9 (CH_2), 46.4 (C^q), 31.0, 28.8 (CH_3), 28.6 (CH), 26.6, 24.9 (CH_3). ^{11}B NMR (160.5 MHz, CD_2Cl_2): δ = –18.9 (br m) ppm. LIFDI-MS (*m/z*) calculated for $[C_{40}H_{64}B_1N_2]$ = 583.5157; found: 583.5142.

[(cAAC^{Me}-H)BH$_2$(pyr)] (5)

Compound **1** (150 mg, 501 µmol) was dissolved in pyridine (1.0 mL) and the reaction mixture stirred at rt for 24 h. After removal of volatiles in vacuo, the resulting pale yellow solid was redissolved in THF (2.0 mL) and cooled to –30 °C. Crystallization afforded **5** as a pale yellow crystalline solid (182 mg, 481 µmol, 96%). $^1H\{^{11}B\}$ NMR (500 MHz, C_6D_6): δ = 7.67 (dm, ^3J = 7.6 Hz, 2H, pyr*H*), 7.35 (dd, ^3J = 7.6 Hz, ^4J = 1.7 Hz, 1H, Ar*H*), 7.19 (t, ^3J = 7.6 Hz, 1H, Ar*H*), 6.94 (dd, ^3J = 7.6 Hz, ^4J = 1.7 Hz, 1H, Ar*H*), 6.51 (tt, ^3J = 7.7 Hz, ^4J = 1.4 Hz, 1H, pyr*H*), 6.15 (ddm, ^3J = 7.6, 6.6 Hz, 2H, pyr*H*), 4.57 (sept, ^3J = 6.8 Hz, 1H, CH_{iPr}), 3.52 (sept, ^3J = 6.8 Hz, 1H, CH_{iPr}), 3.24 (br t, ^3J = 3.5 Hz, 1H, BC*H*), 3.00 (br s, 2H, BH_2), 2.07–1.99 (2 x d, ^2J = 12.3 Hz, 2H, CH_2), 1.71 (d, ^3J = 6.8 Hz, 3H, $CH_{3\text{-iPr}}$), 1.67 (s, 3H, CH_3), 1.52 (s, 3H, CH_3), 1.48 (d, ^3J = 6.8 Hz, 3H, $CH_{3\text{-iPr}}$), 1.29–1.24 (overlapping s, 3H, CH_3 and d, 3H, $CH_{3\text{-iPr}}$), 1.11 (s, 3H, CH_3), 0.55 (d, ^3J = 6.8 Hz, 3H, $CH_{3\text{-iPr}}$) ppm. $^{13}C\{^1H\}$ NMR (125.8 MHz, C_6D_6): δ = 243.5 ($C_{carbene}$, HMBC), 153.7, 152.4 (C^q), 147.5 (CH_{Ar}), 143.4 (C^q), 137.8, 125.9, 124.7, 124.6, 124.4, 147.5 (CH_{Ar}), 74.0 (CH), 62.0 (C^q), 60.6 (CH_2), 41.1 (C^q), 32.0, 30.0, 29.7 (CH_3), 29.2, 27.7 (CH), 26.6, 26.5, 26.1, 25.7, 24.3 (CH_3). ^{11}B NMR (160.5 MHz, C_6D_6): δ = –4.41 (br) ppm. LIFDI-MS (*m/z*) calculated for $[C_{25}H_{38}B_1N_2]$ = 377.3123; found: 377.3115.

NMR Spectra

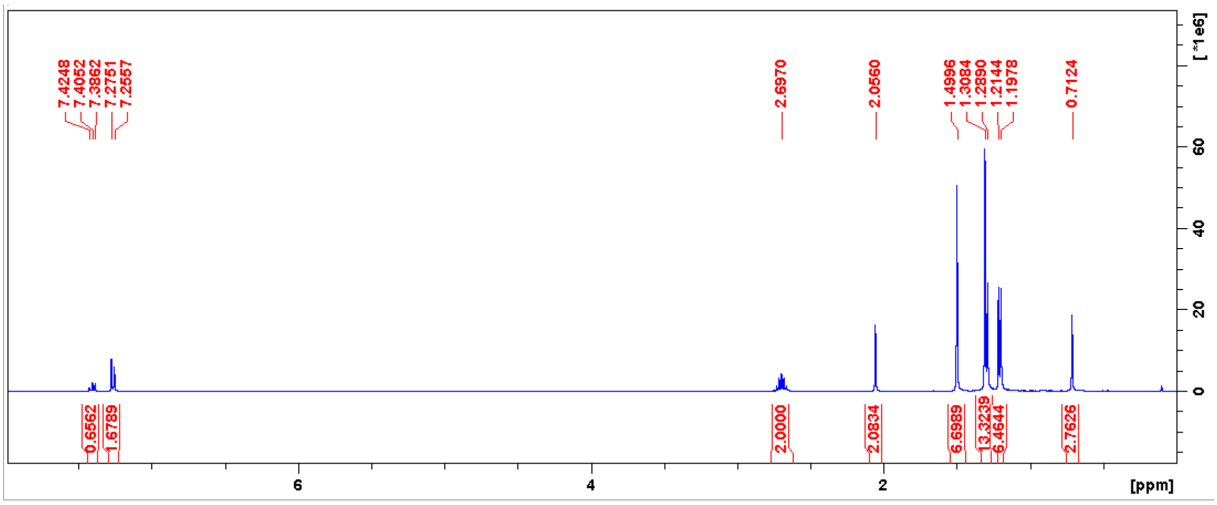

Figure S1. $^{1}H\{^{11}B\}$ NMR spectrum of **1** in CD_2Cl_2.

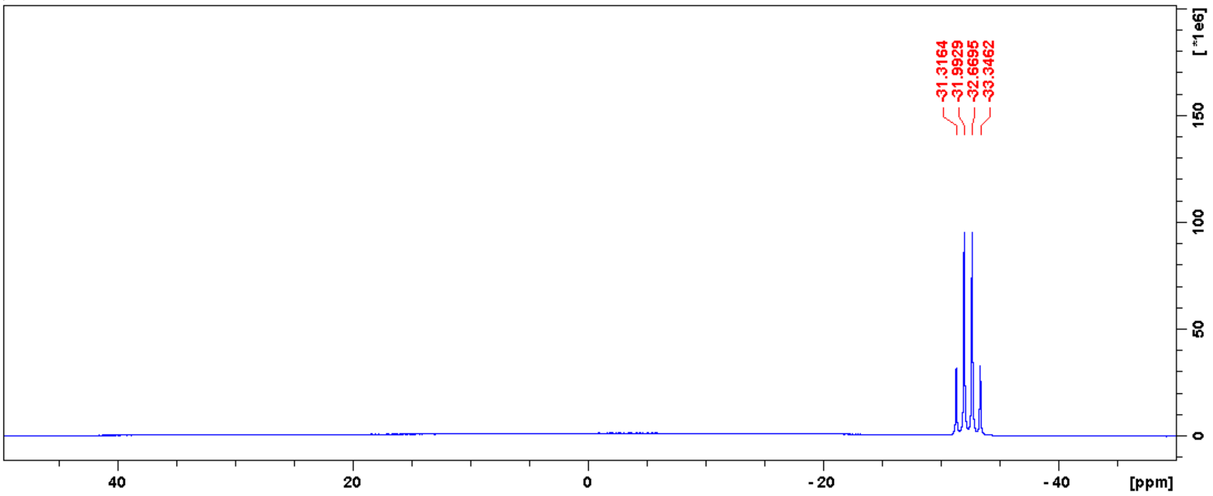

Figure S2. ^{11}B NMR spectrum of **1** in CD_2Cl_2.

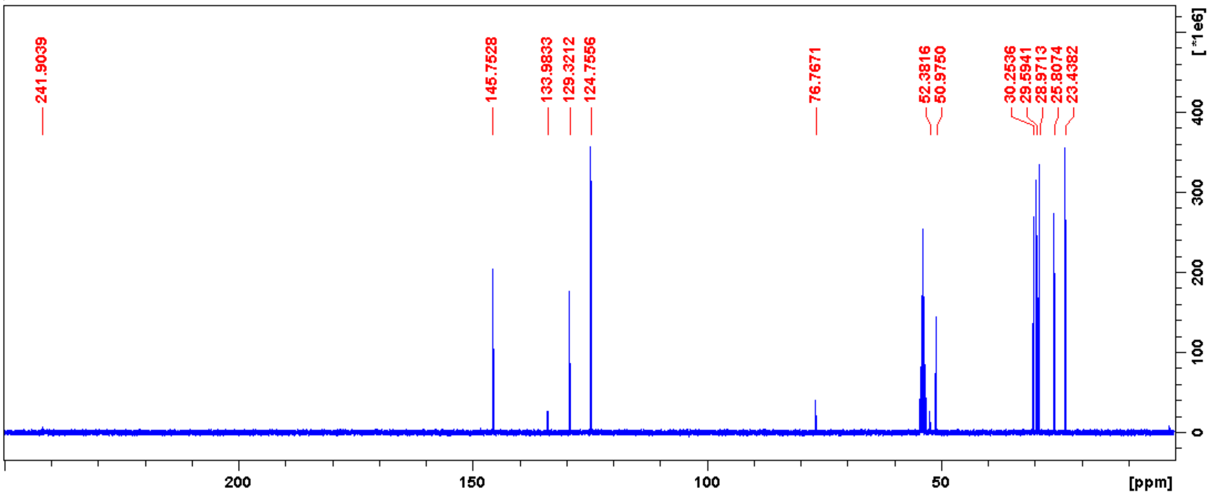

Figure S3. $^{13}C\{^{1}H\}$ NMR spectrum of **1** in CD_2Cl_2.

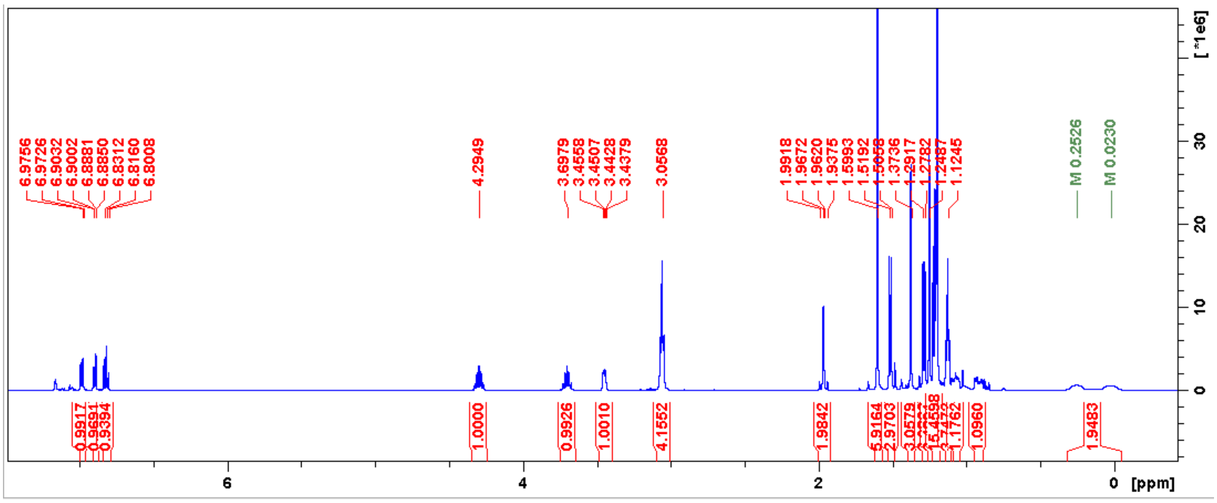

Figure S4. $^{1}H\{^{11}B\}$ NMR spectrum of **2a** in C_6D_6.

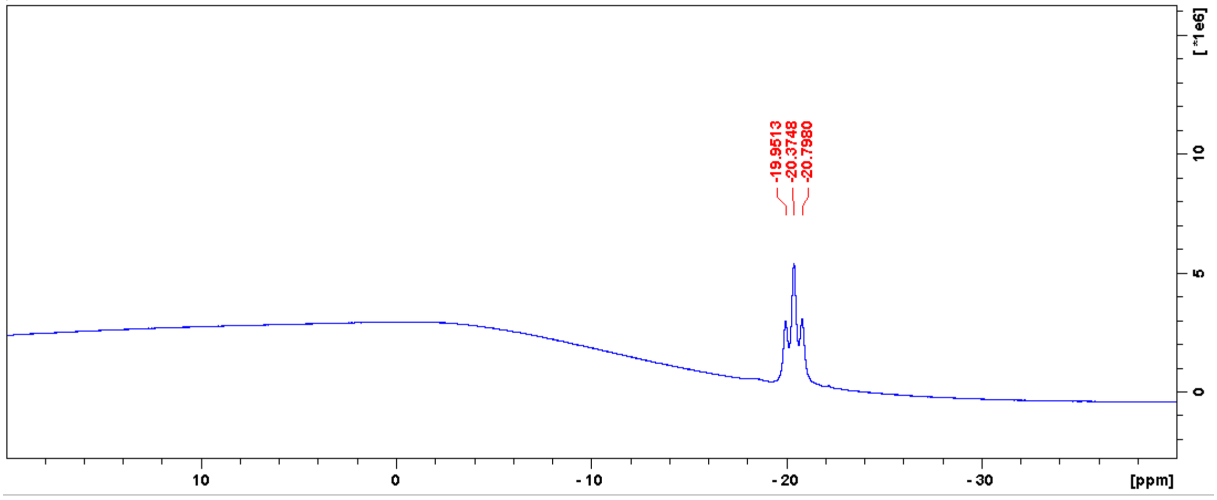

Figure S5. ^{11}B NMR spectrum of **2a** in C_6D_6.

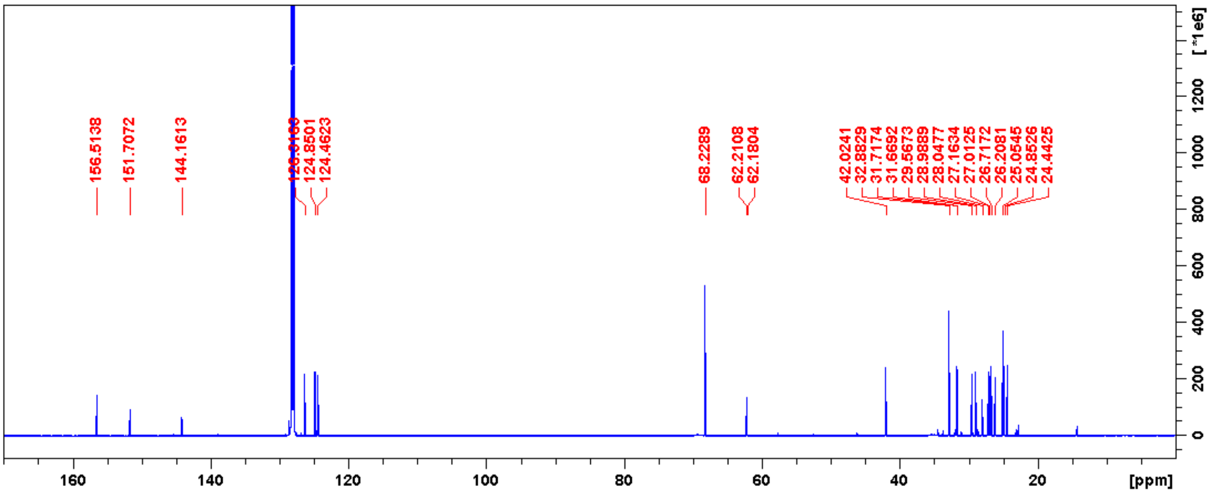

Figure S6. $^{13}C\{^{1}H\}$ NMR spectrum of **2a** in C_6D_6.

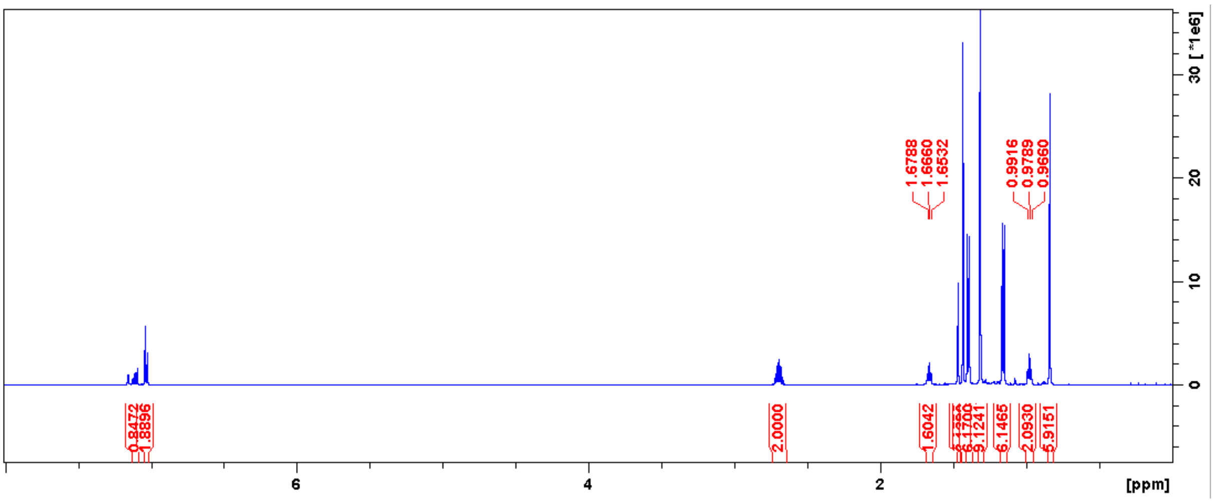

Figure S7. $^{1}H\{^{11}B\}$ NMR spectrum of **3a** in C_6D_6.

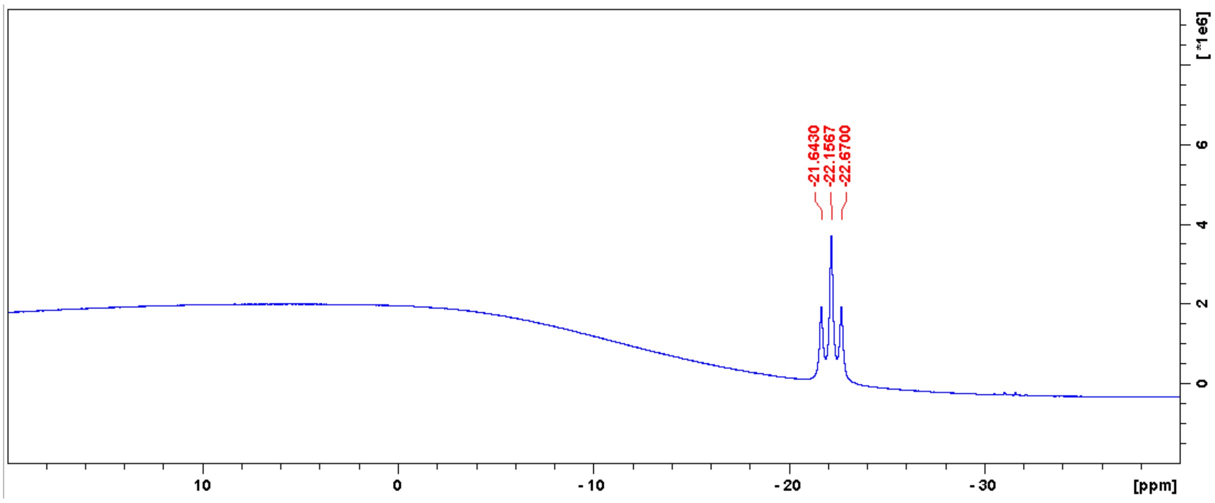

Figure S8. ^{11}B NMR spectrum of **3a** in C_6D_6.

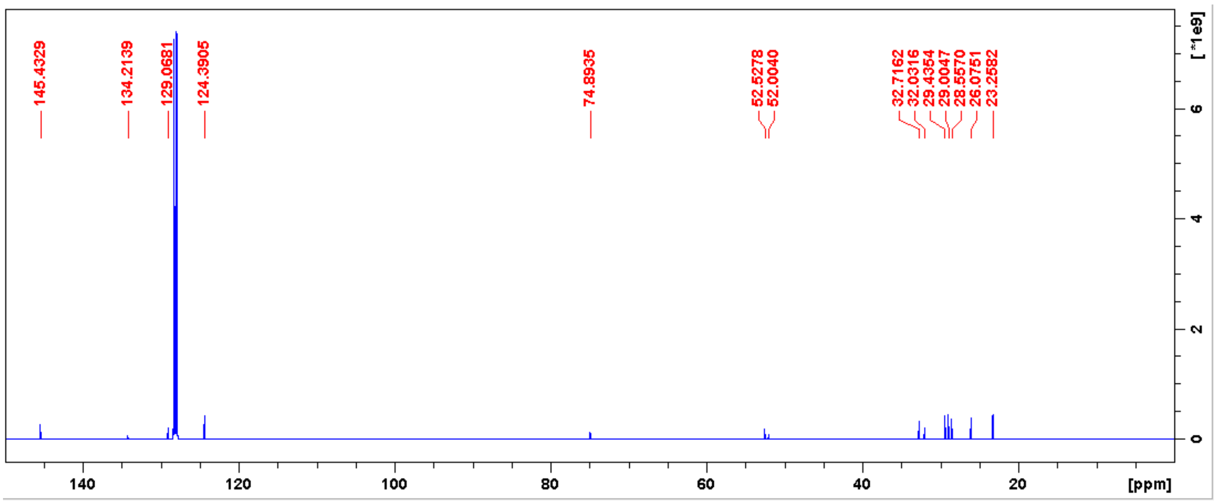

Figure S9. $^{13}C\{^{1}H\}$ NMR spectrum of **3a** in C_6D_6.

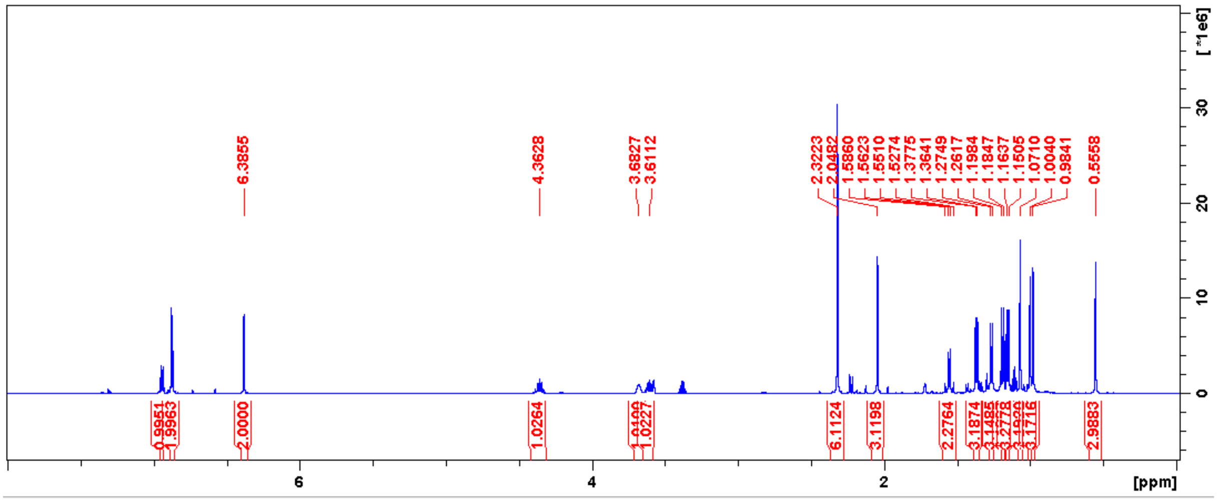

Figure S10. $^{1}H\{^{11}B\}$ NMR spectrum of **2b** in d_8-THF.

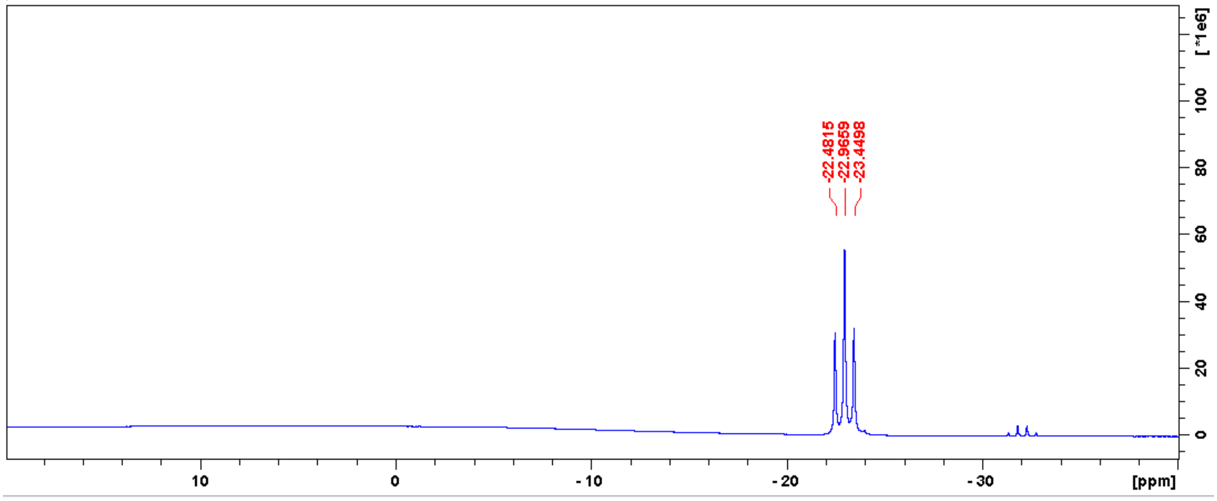

Figure S11. ^{11}B NMR spectrum of **2b** in d_8-THF.

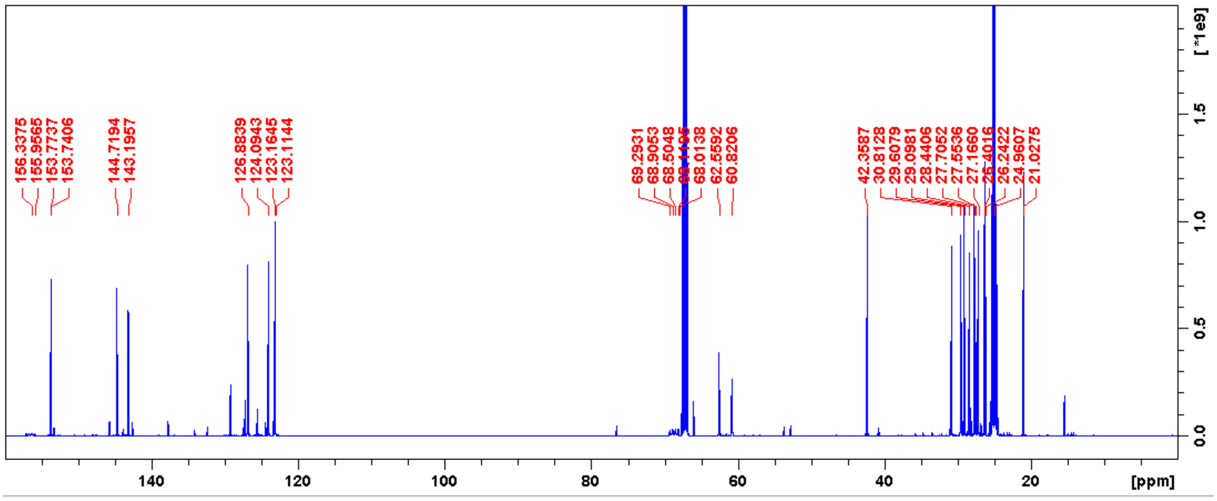

Figure S12. $^{13}C\{^{1}H\}$ NMR spectrum of **2b** in d_8-THF.

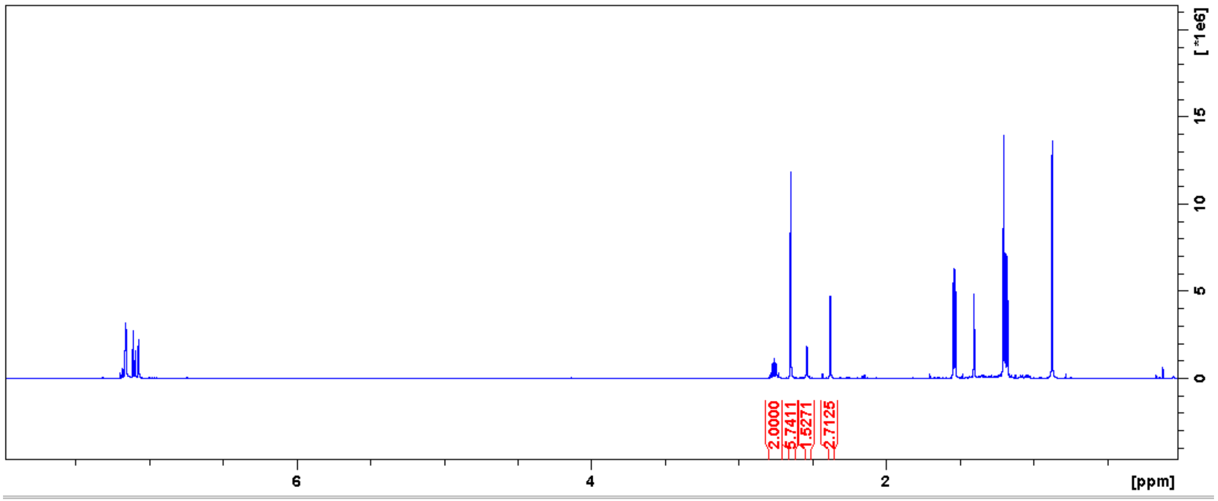

Figure S13. $^{1}H\{^{11}B\}$ NMR spectrum of **3b** in C_6D_6.

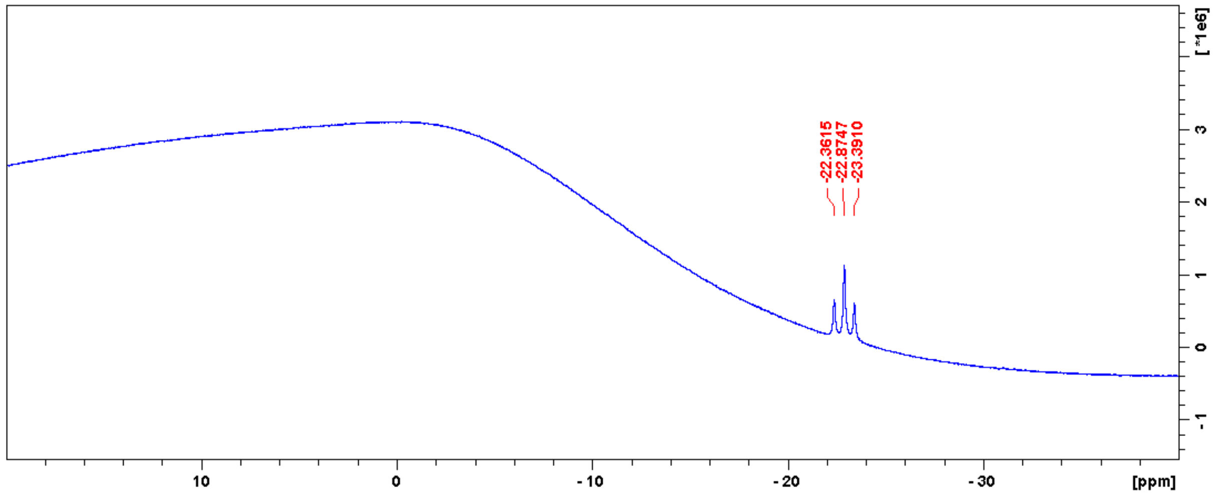

Figure S14. ^{11}B NMR spectrum of **3b** in C_6D_6.

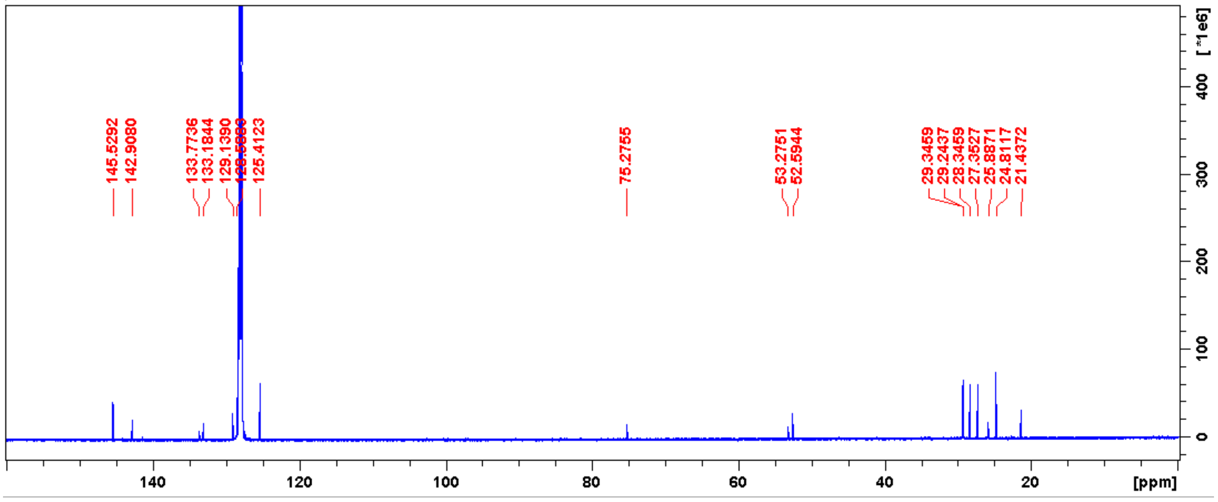

Figure S15. $^{13}C\{^{1}H\}$ NMR spectrum of **3b** in C_6D_6.

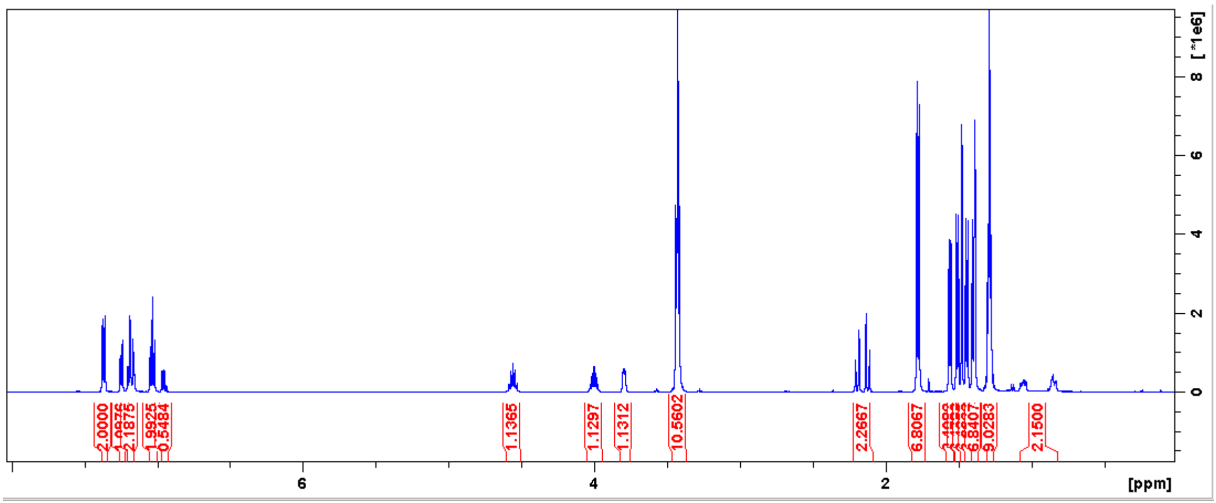

Figure S16. $^{1}H\{^{11}B\}$ NMR spectrum of **2c** in C_6D_6.

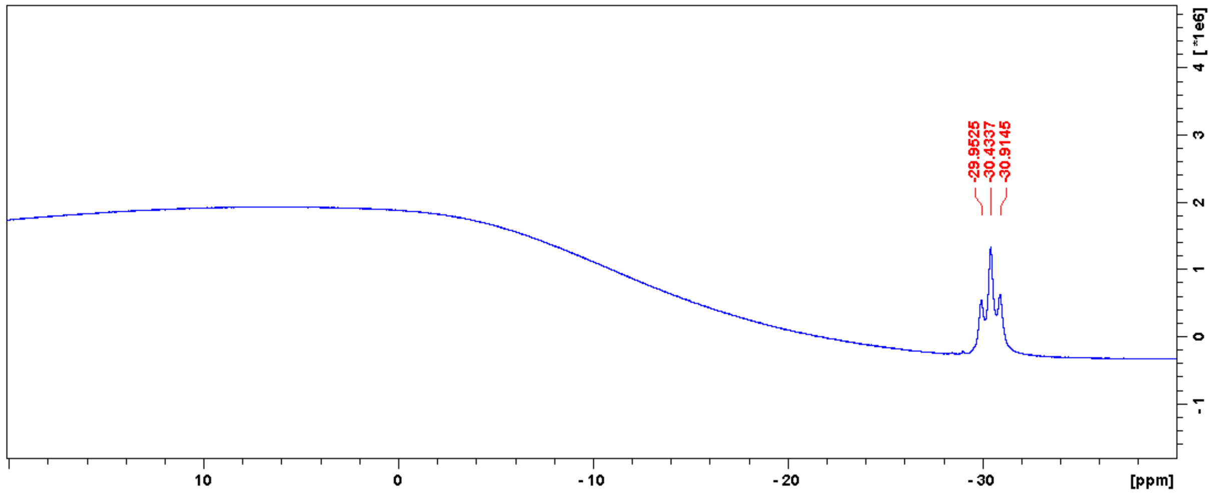

Figure S17. ^{11}B NMR spectrum of **2c** in C_6D_6.

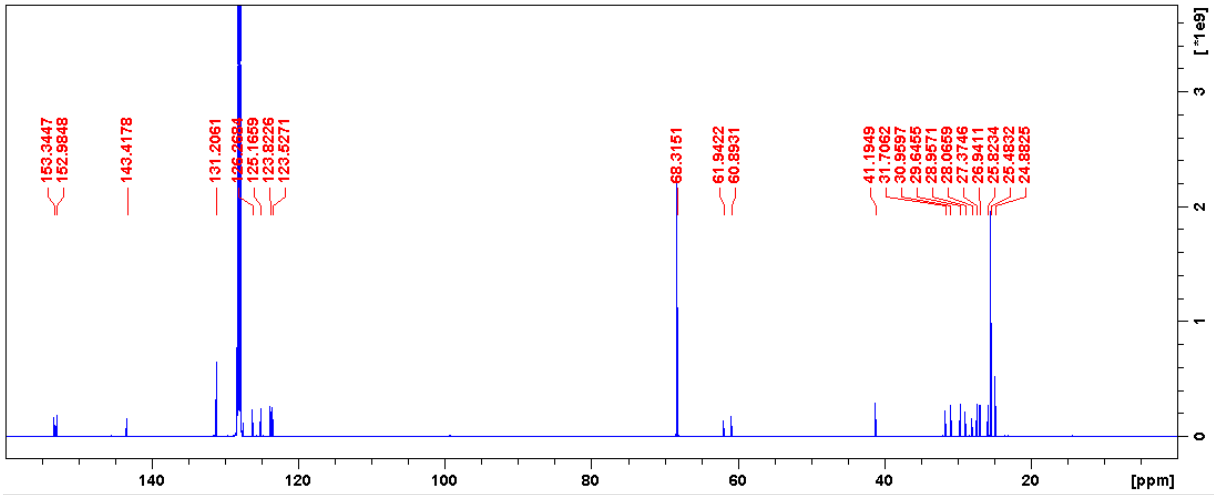

Figure S18. $^{13}C\{^{1}H\}$ NMR spectrum of **2c** in C_6D_6.

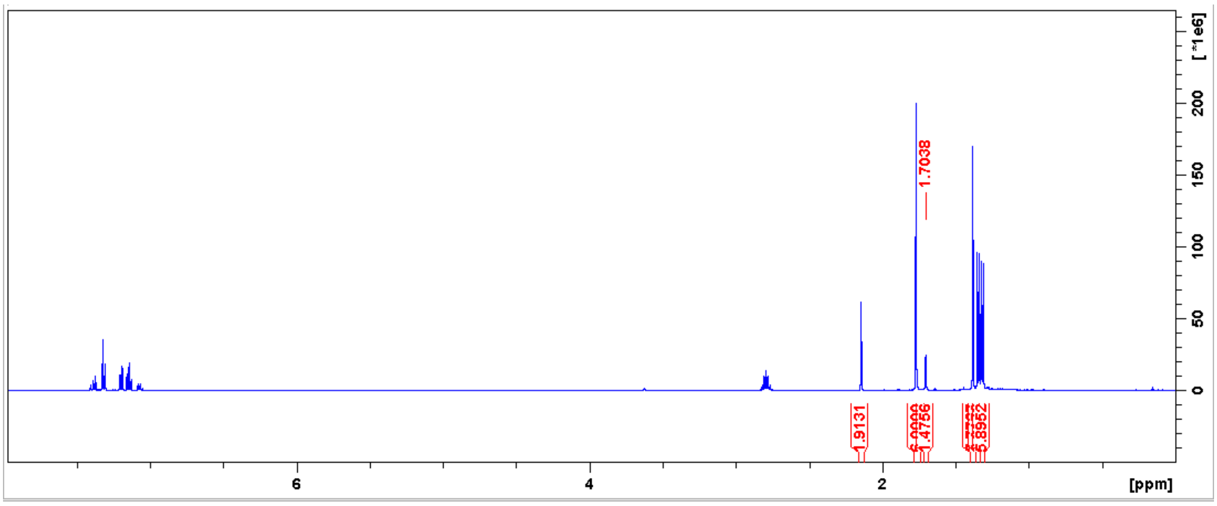

Figure S19. $^{1}H\{^{11}B\}$ NMR spectrum of **3c** in C_6D_6.

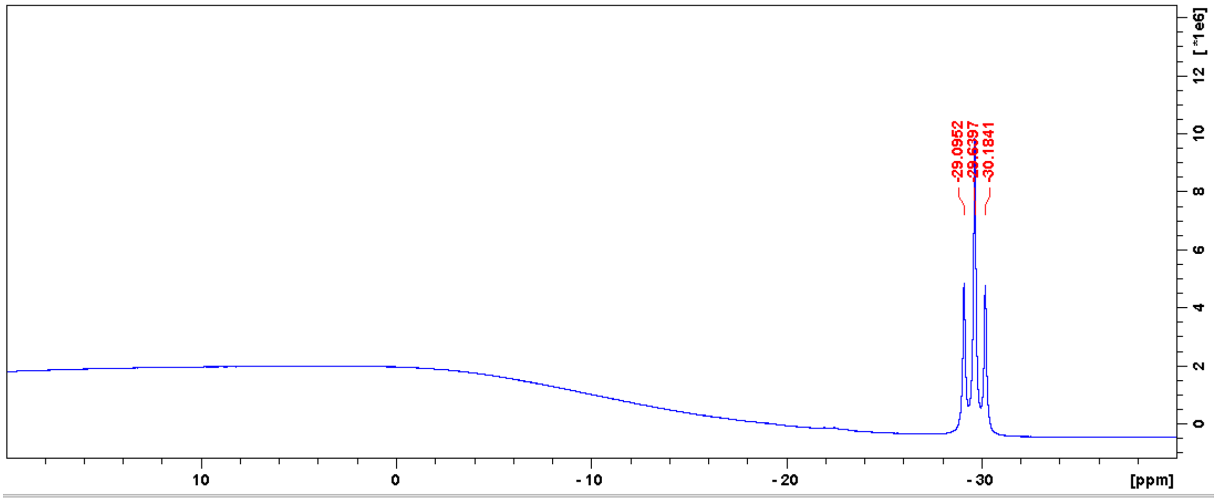

Figure S20. ^{11}B NMR spectrum of **3c** in C_6D_6.

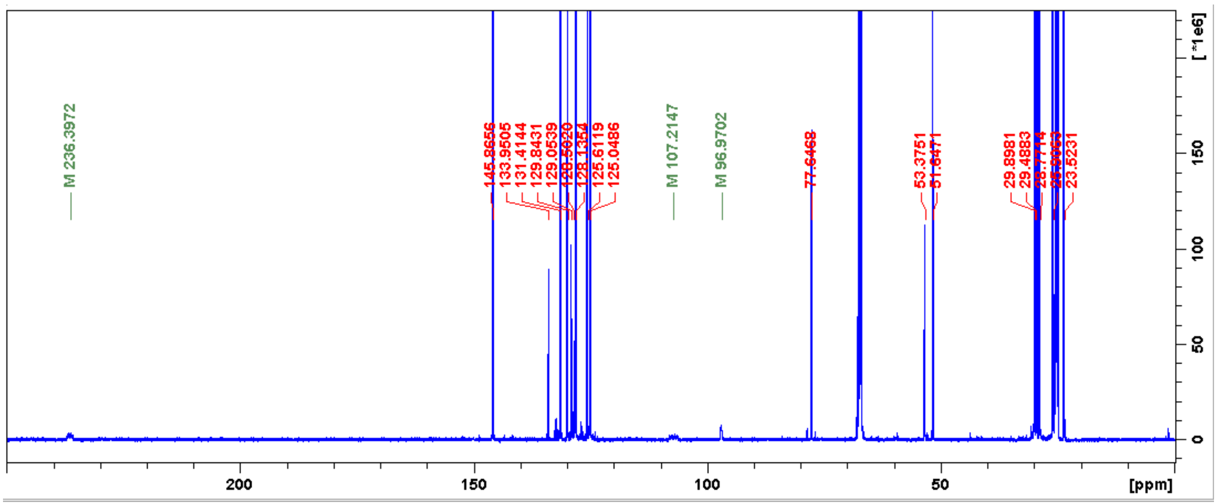

Figure S21. $^{13}C\{^{1}H\}$ NMR spectrum of **3c** in C_6D_6.

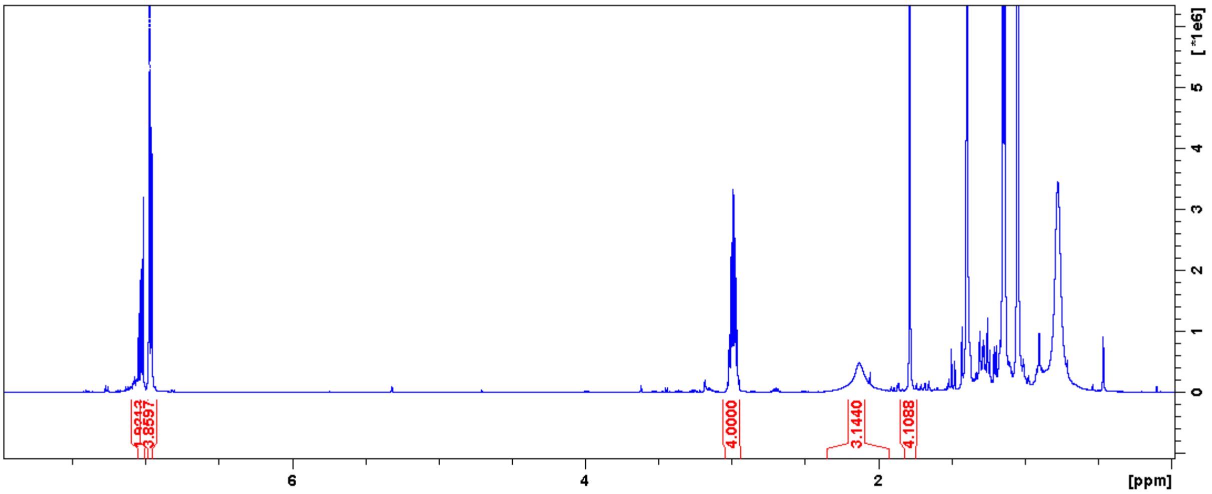

Figure S22. $^{1}H\{^{11}B\}$ NMR spectrum of **4** in CD_2Cl_2 at rt.

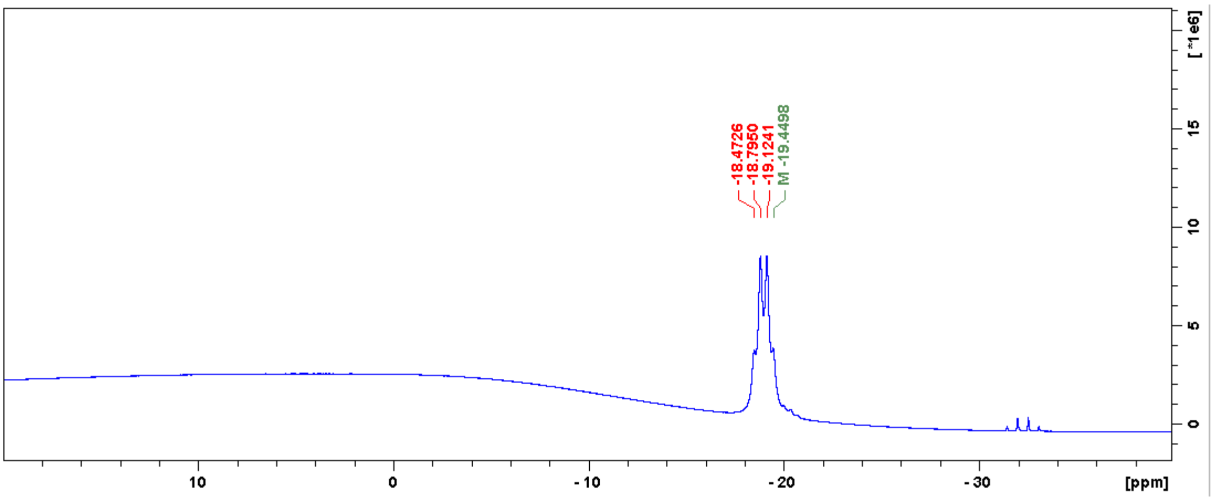

Figure S23. ^{11}B NMR spectrum of **4** in CD_2Cl_2 at rt.

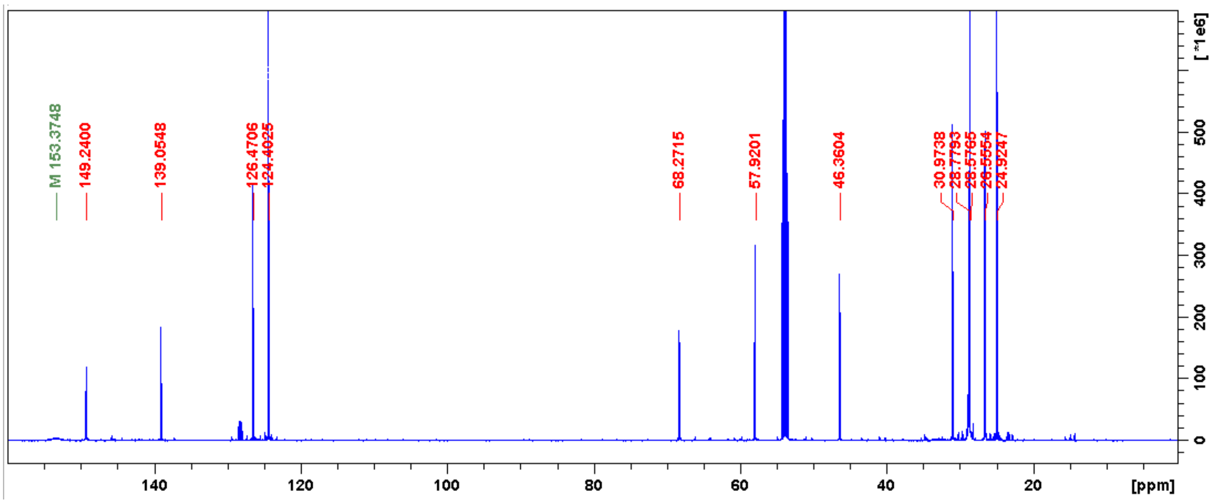

Figure S24. $^{13}C\{^{1}H\}$ NMR spectrum of **4** in CD_2Cl_2 at rt.

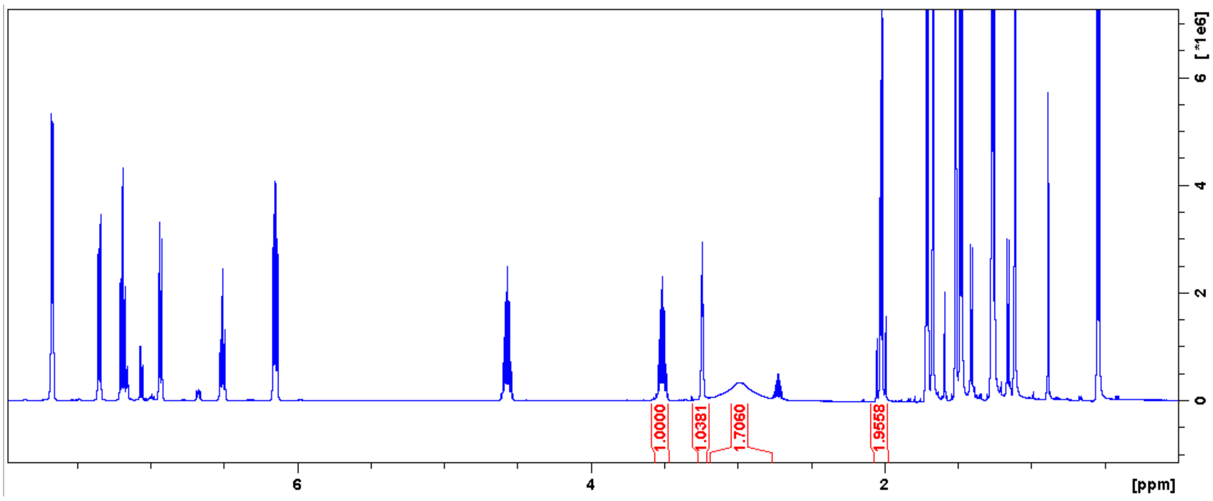

Figure S25. $^{1}H\{^{11}B\}$ NMR spectrum of **5** in C_6D_6 (ca. 10% **1** + free pyridine in solution).

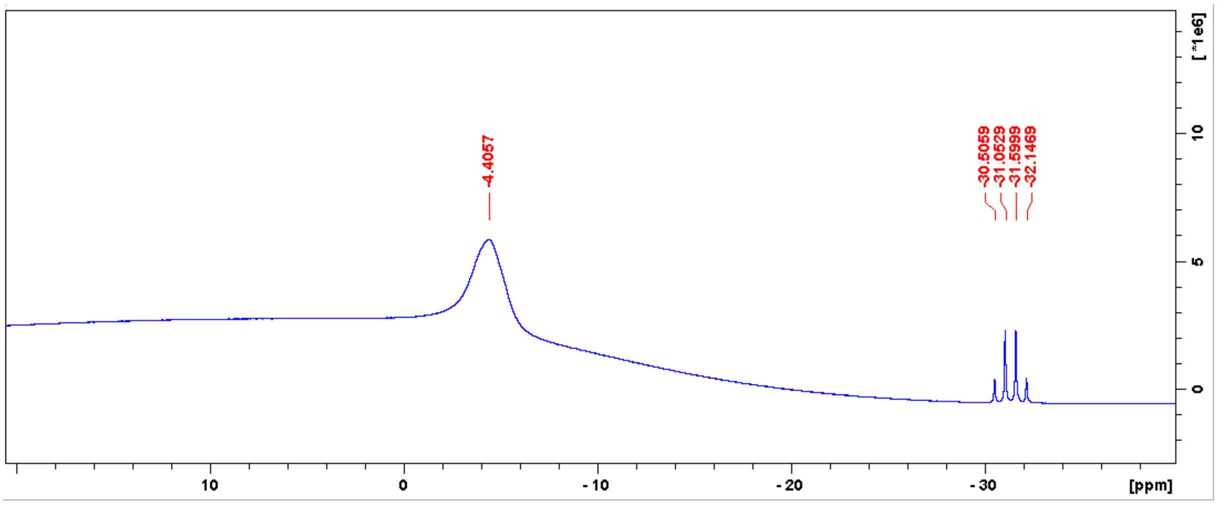

Figure S26. ^{11}B NMR spectrum of **5** in C_6D_6 (ca. 10% **1** + free pyridine in solution).

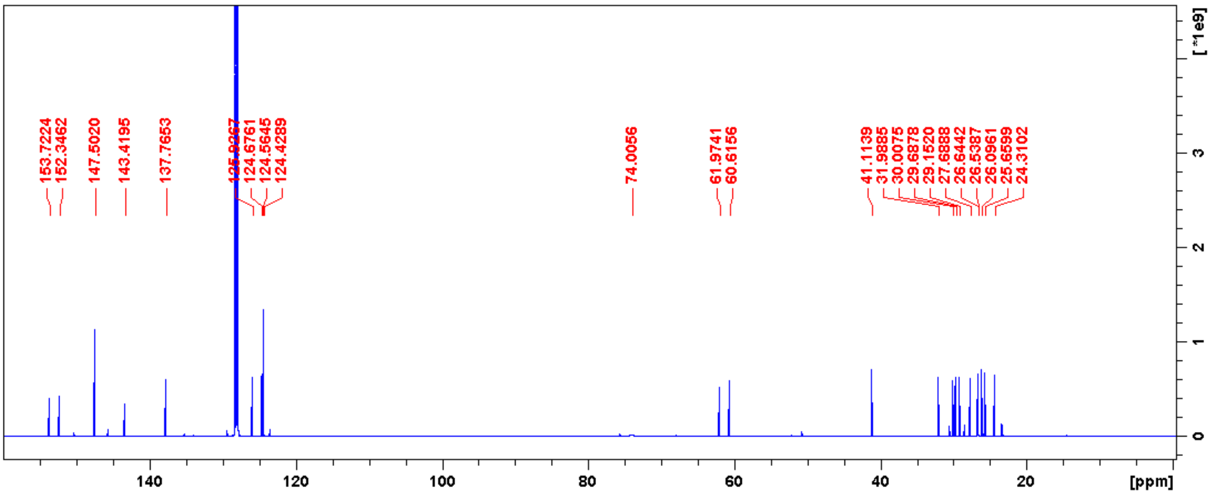

Figure S27. $^{13}C\{^{1}H\}$ NMR spectrum of **5** in C_6D_6 (ca. 10% **1** + free pyridine in solution).

Low temperature NMR study of 4

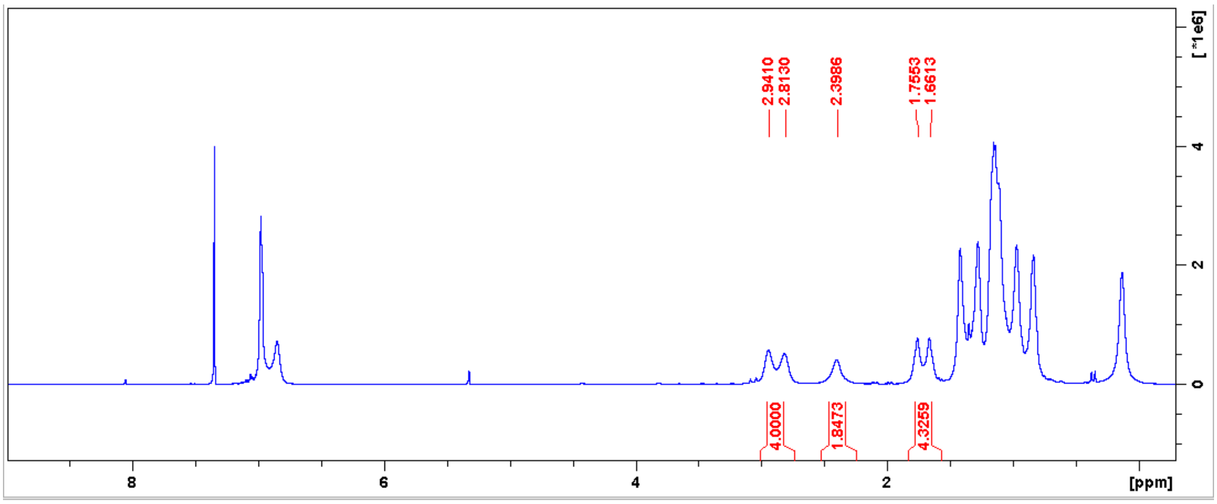

Figure S28. $^{1}H\{^{11}B\}$ NMR spectrum of **4** in in CD_2Cl_2 at –40 °C.

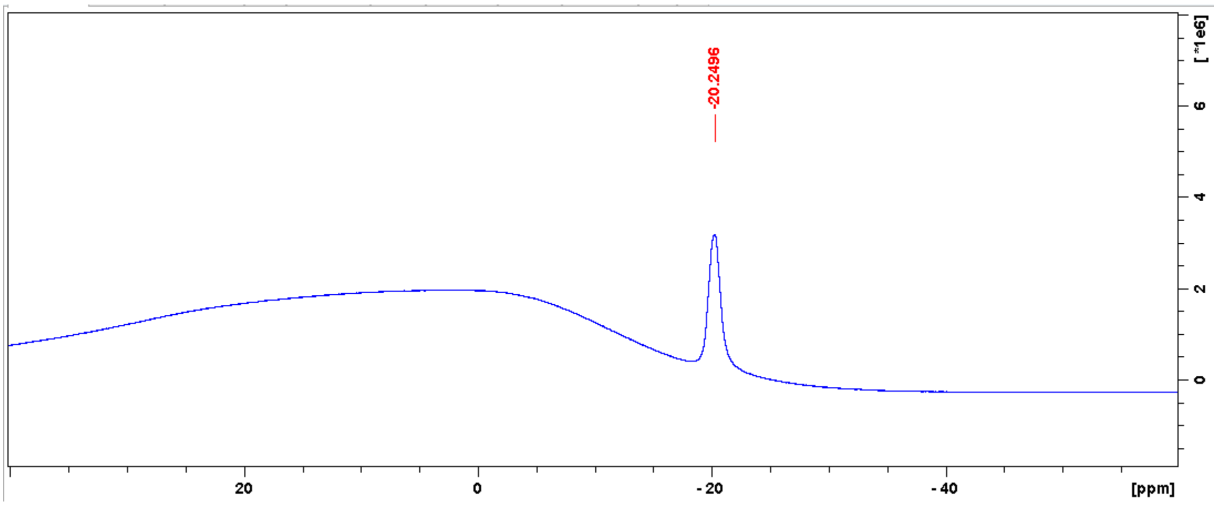

Figure S29. ^{11}B NMR spectrum of **4** in in CD_2Cl_2 at –40 °C.

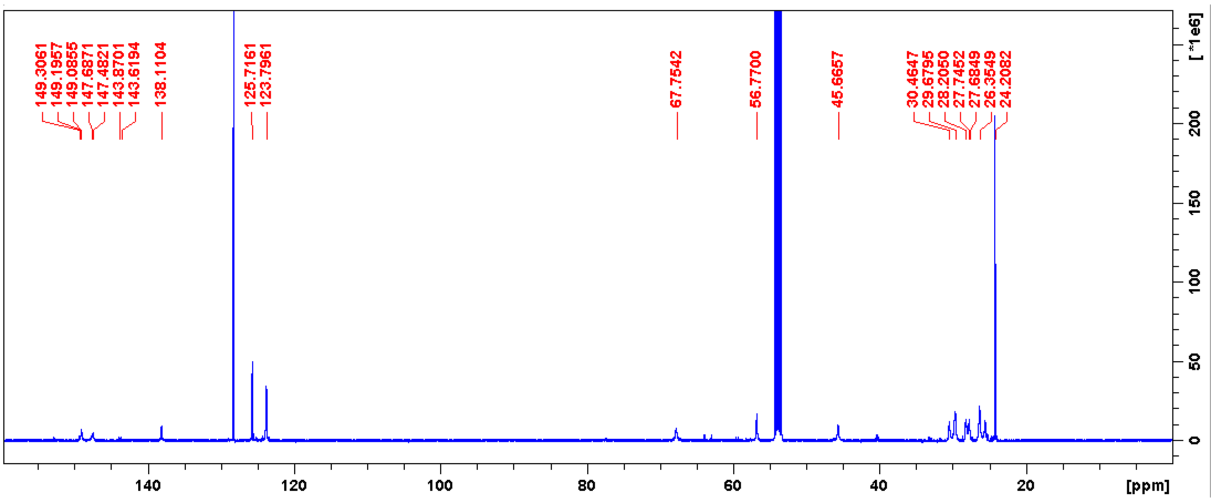

Figure S30. $^{13}C\{^{1}H\}$ NMR spectrum of **4** in in CD_2Cl_2 at –40 °C.

Variable-Temperature NMR Study of 5 in C_6D_6 (0.11 M)

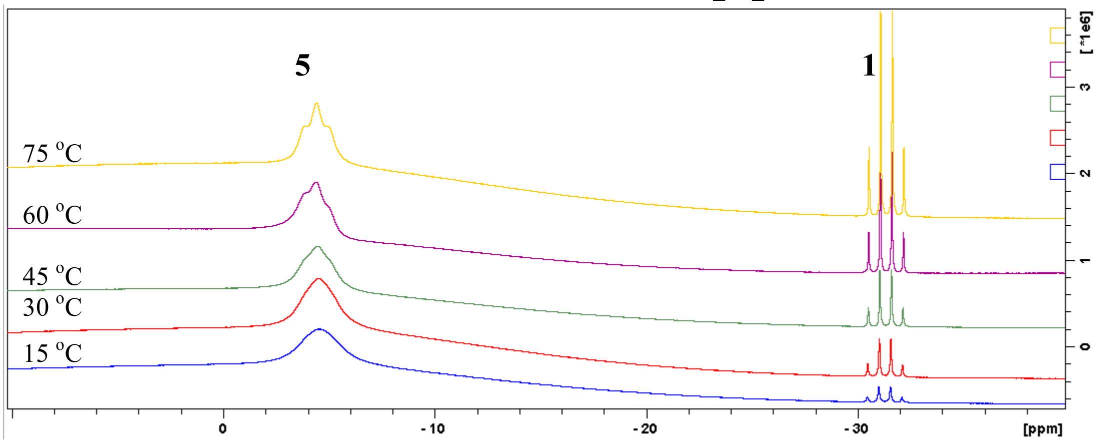

Figure S31. Temperature stack-plot of ^{11}B NMR spectra of a C_6D_6 solution of **5**.

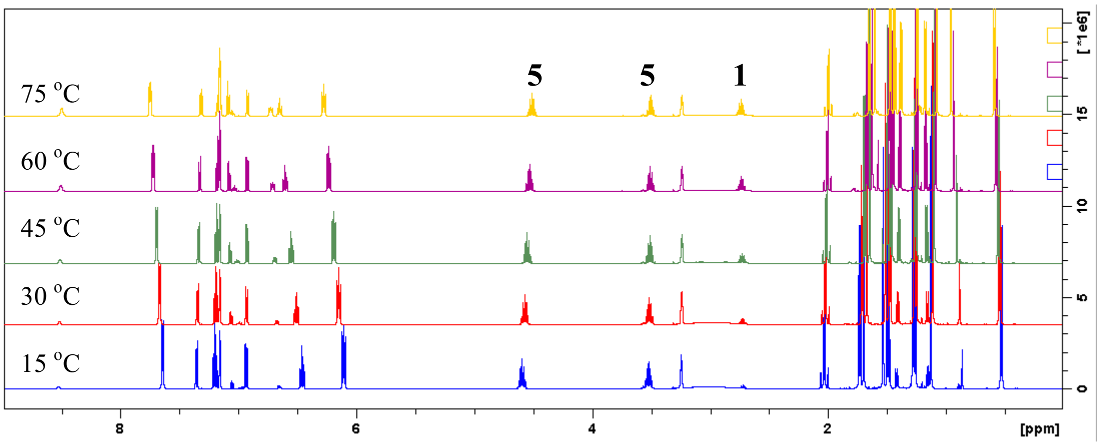

Figure S32. Temperature stack-plot of ^{1}H NMR spectra of a C_6D_6 solution of **5**.

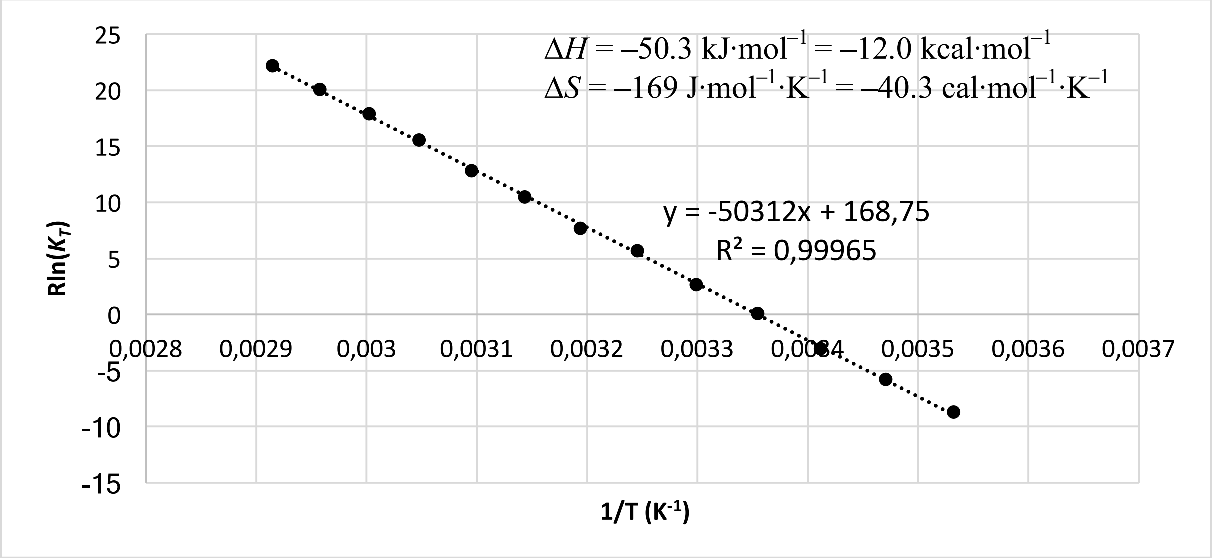

Figure S33. Van’t Hoff analysis of the equilibrium **1** + pyr $\underset{-\Delta G(\mathrm{T})}{\overset{\Delta G(\mathrm{T})}{\rightleftharpoons}}$ **5**

Crystallographic Details

Crystal data were collected on a Bruker X8-APEX II diffractometer with a CCD area detector and multi-layer mirror monochromated $Mo_{K\alpha}$ radiation or on a Bruker D8 Quest with a Photon100 detector. The structures were solved using direct methods, refined with the ShelX software package and expanded using Fourier techniques.[5] All non-hydrogen atoms were refined anisotropically. Hydrogen atoms were assigned to idealized positions, with the exception of the boron-bound hydrides, which were all located in the difference Fourier map and freely refined. Figures of the crystallographically-determined solid-state structures of all compounds were made using Mercury freeware[6] in combination with PovRay.[7]

Refinement details for 3b: The asymmetric unit contains two distinct molecules of **3b**. The second molecule, centered on B2, presents a twofold disorder in the cAAC backbone (C33 > C35, C37, C38) modelled in a 93:7 ratio. As a result, the ADPs of these atoms were restrained to be similar to C32 and C36 using SIMU 0.002 and the C36-C37B/C38B bond length was restrained to be similar to C36-C37A. To avoid H-H bumping into the PART -1, the distance between H41 and H34E was restrained to be greater than 2.2 Å.

Refinement details for 2c: The structure contained three Li-bound THF molecules, two of which were twofold disordered in the carbon atoms in a ratio of 89:11 and 46:54, respectively, the third being threefold disordered in its carbon atoms in a 40:36:24 ratio. All THF atom ADPs were restrained using SIMU 0.005.

Refinement details for 4: The asymmetric unit contains half a CH_2Cl_2 molecule positioned on the inversion center. The Cl atoms were modelled with a 59:42 rotation disorder around the methylene carbon. The ADPs of the Cl atoms were restricted with EADP.

Crystal data for 1. Formula: $C_{20}H_{34}BN$, $M_r = 299.29$, colorless block, 0.492 x 0.122 x 0.100 mm^3, monoclinic space group $P\ 2_1/n$, $a = 9.4869(7)$ Å, $b = 16.4069(12)$ Å, $c = 12.2003(8)$ Å, $\beta = 93.931(2)°$, $V = 1894.5(2)$ Å^3, $Z = 4$, $\rho_{calcd} = 1.049$ g·cm^{-3}, $\mu = 0.059$ mm^{-1}, $F(000) = 664$, $T = 103(2)$ K, $R_1 = 0.0411$, $wR^2 = 0.1110$, $R_{int} = 0.0534$, 4532 independent reflections [2θ≤55.944°] and 219 parameters.

Crystal data for 2a. Formula: $C_{29}H_{53}BLiNO$, $M_r = 449.47$, colorless block, 0.273 x 0.247 x 0.244 mm^3, monoclinic space group $P\ 2_1/n$, $a = 9.633(2)$ Å, $b = 31.696(7)$ Å, $c = 10.432(2)$ Å, $\beta = 115.215(6)°$, $V = 2881.6(11)$ Å^3, $Z = 4$, $\rho_{calcd} = 1.036$ g·cm^{-3}, $\mu = 0.059$ mm^{-1}, $F(000) = 1000$, $T = 100(2)$ K, $R_1 = 0.0568$, $wR^2 = 0.1432$, $R_{int} = 0.0513$, 6413 independent reflections [2θ≤54.43°] and 317 parameters.

Crystal data for 3a. Formula: $C_{25}H_{44}BN$, $M_r = 369.42$, colorless block, 0.464 x 0.364 x 0.332 mm^3, monoclinic space group $P\ 2_1/n$, $a = 15.4830(17)$ Å, $b = 9.8194(10)$ Å, $c = 16.6483(19)$ Å, $\beta = 105.121(4)°$, $V = 2443.5(5)$ Å^3, $Z = 4$, $\rho_{calcd} = 1.004$ g·cm^{-3}, $\mu = 0.056$ mm^{-1}, $F(000) = 824$, $T = 100(2)$ K, $R_1 = 0.0469$, $wR^2 = 0.1100$, $R_{int} = 0.0751$, 5369 independent reflections [2θ≤54.18°] and 263 parameters.

Crystal data for 3b. Formula: $C_{29}H_{44}BN$, M_r = 417.46, colorless block, 0.674 x 0.354 x 0.156 mm^3, monoclinic space group $P\ 2_1/n$, a = 13.724(3) Å, b = 16.513(3) Å, c = 23.647(5) Å, β = 105.61(3)°, V = 5161(2) Å^3, Z = 8, ρ_{calcd} = 1.075 g·cm^{-3}, μ = 0.060 mm^{-1}, F(000) = 1840, T = 100(2) K, R_1 = 0.0663, wR^2 = 0.1884, R_{int} = 0.0874, 12282 independent reflections [2θ≤55.194°] and 647 parameters.

Crystal data for 2c. Formula: $C_{40}H_{63}BLiNO_3$, M_r = 623.70, colorless block, 0.557 x 0.428 x 0.369 mm^3, orthorhombic space group P b c a, a = 17.9286(9) Å, b = 17.5371(9) Å, c = 24.5618(13) Å, V = 7722.6(7) Å^3, Z = 8, ρ_{calcd} = 1.073 g·cm^{-3}, μ = 0.065 mm^{-1}, F(000) = 2736, T = 100(2) K, R_1 = 0.0531, wR^2 = 0.1275, R_{int} = 0.0763, 8557 independent reflections [2θ≤54.43°] and 580 parameters.

Crystal data for 4. Formula: $C_{40}H_{65}BN_2(CH_2Cl_2)_{0.5}$, M_r = 627.21, colorless block, 0.560 x 0.350 x 0.300 mm^3, triclinic space group $P\bar{1}$, a = 9.0886(6) Å, b = 11.7351(10) Å, c = 18.4941(15) Å, α = 82.046(4)°, β = 75.867(2)°, γ = 89.921(3)°, V = 1893.3(3) Å^3, Z = 2, ρ_{calcd} = 1.100 g·cm^{-3}, μ = 0.130 mm^{-1}, F(000) = 690, T = 100(2) K, R_1 = 0.0645, wR^2 = 0.1815, R_{int} = 0.1406, 8312 independent reflections [2θ≤54.306°] and 442 parameters.

Crystal data for 5. Formula: $C_{25}H_{39}BN_2$, M_r = 378.39, colorless block, 0.677 x 0.282 x 0.156 mm^3, orthorhombic space group Pbca, a = 15.5756(10) Å, b = 16.3913(10) Å, c = 18.0729(11) Å, V = 4614.1(5) Å^3, Z = 8, ρ_{calcd} = 1.100 g·cm^{-3}, μ = 0.062 mm^{-1}, F(000) = 690, T = 100(2) K, R_1 = 0.0466, wR^2 = 0.1295, R_{int} = 0.0854, 5097 independent reflections [2θ≤54.266°] and 270 parameters.

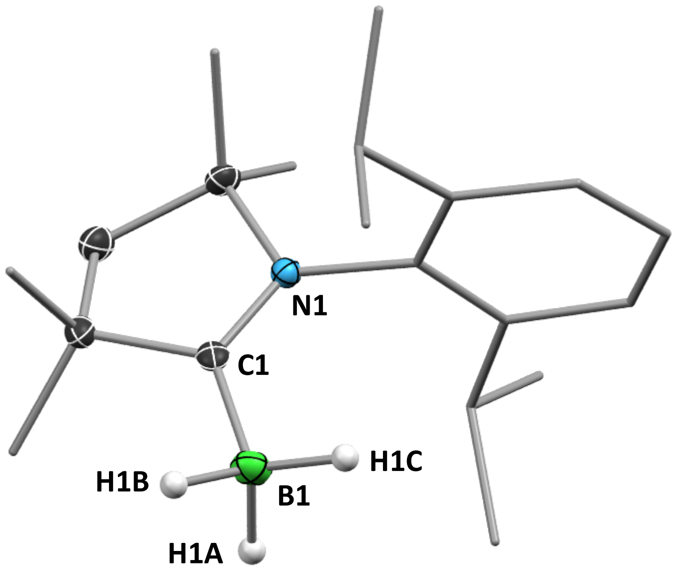

Figure S34. Crystallographically-determined solid-state structure of **1**. Atomic displacement ellipsoids depicted at 50% probability level and omitted for the ligand periphery. Hydrogen atoms omitted except for those bound to B1. Selected bond lengths (Å): B1-C1 1.5822(14), C1-N1 1.3076(12), B1-H1A 1.151(15), B1-H1B 1.136(16), B1-H1C 1.113(14).

Computational Details

All the static DFT calculations were performed with the Gaussian 09 set of programs[8] by using a two-layer ONIOM scheme developed by Morokuma and coworkers.[9] Within this scheme, the dip and methyl substituents on the cAAC^{Me} ligands and the tetramethylene fragment of THF molecules were set as a part of the low layer. For the high layer, the meta-GGA M06-2X density-functional from Zhao and Truhlar,[10] which empirically reproduces very well the dispersion effects, was used. The electronic configurations of the molecular systems in this layer were described with a split-valence basis set of triple-ζ quality with one polarization function and a set of diffuse functions, 6-311+G(d), for all the atoms. For the low layer, the semi-empirical method PM6 was employed.[11] Geometry optimizations were carried out without symmetry constraints, and the stationary points were characterized by analytical frequency calculations. The PM6 charges were utilized for the QM part on the model system (embedcharge keyword in Gaussian09). The universal solvation model (SMD),[12] based on the charge density of a solute molecule interacting with a continuum dielectric of the solvent, was also used in the geometry optimizations to model the solvent effects. Tetrahydrofuran was set up as the solvent. The reported Gibbs free energies in this work include zero-point energy, thermal and entropic corrections evaluated at 298 K and 1 atm in the gas phase.

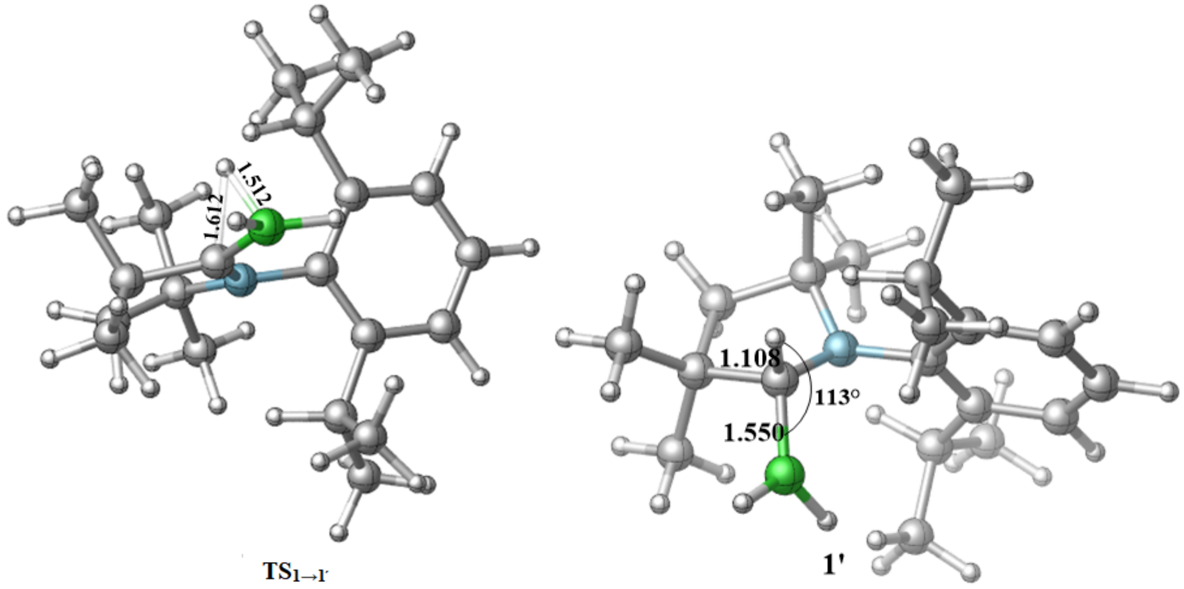

Figure S35. Optimized geometries of the transition state from **1** to its tautomer **1′** and of **1′** itself calculated at the SMD(thf):ONIOM(M06-2X/6-311+G(d):PM6) level. Selected bond distances are in Å.

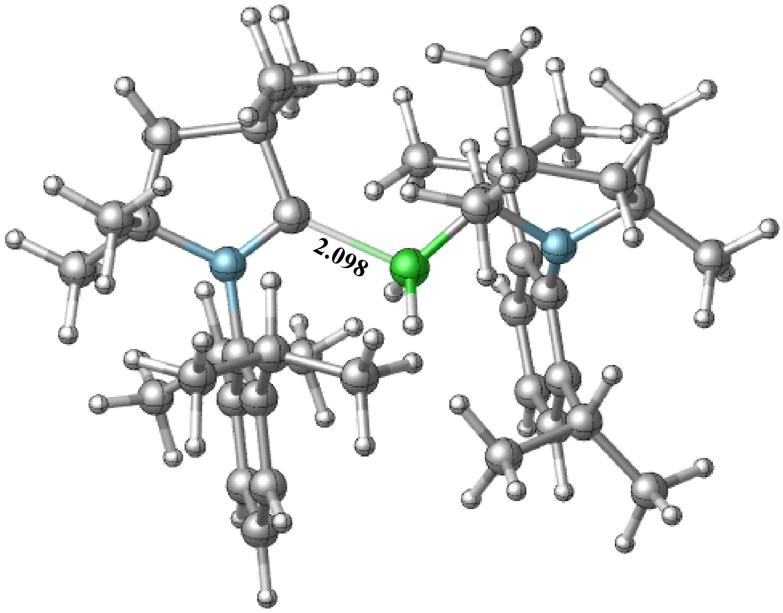

$TS_{A1\rightarrow 4}$

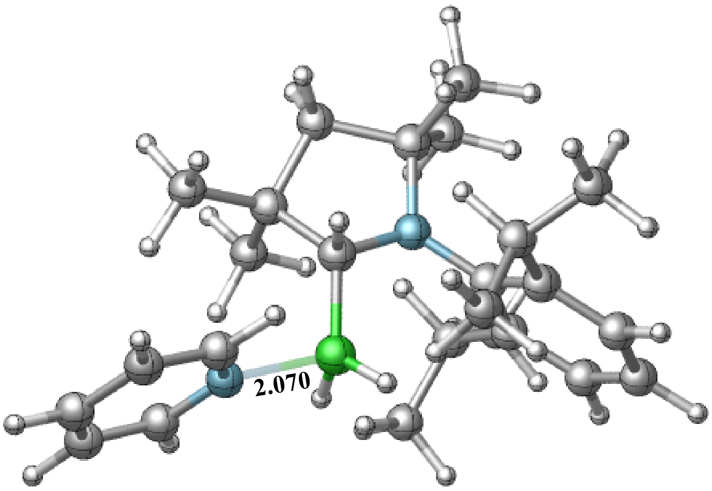

$TS_{A2\rightarrow 5}$

Figure S36. Optimized geometries of the transition states found in the reaction mechanisms calculated at the SMD(thf):ONIOM(M06-2X/6-311+G(d):PM6) level. Selected bond distances are in Å.

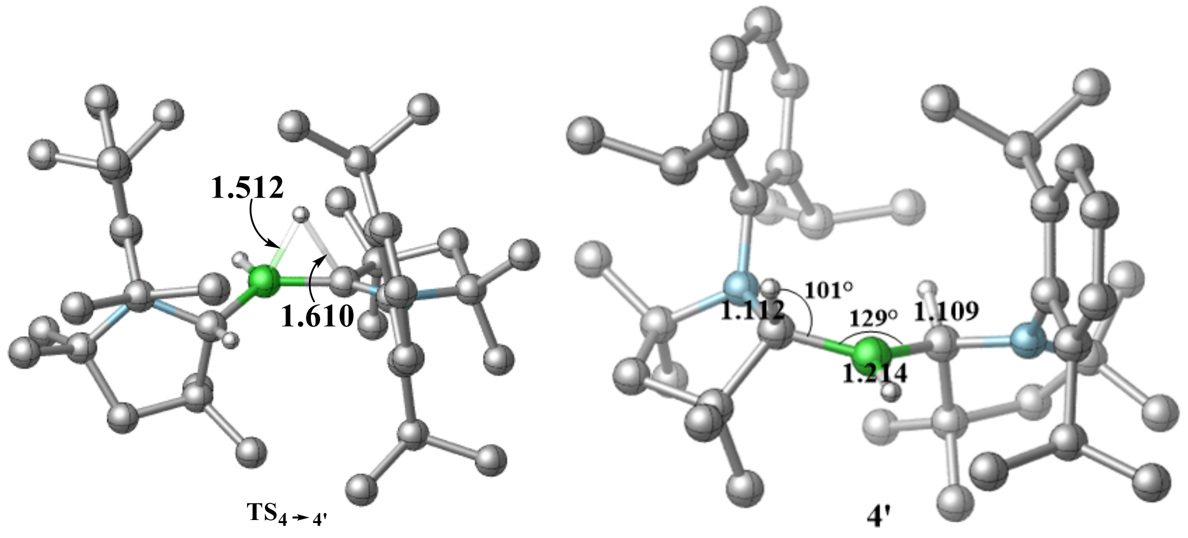

Figure S37. Optimized geometries of the transition state from **4** to its tautomer **4′** and of **4′** itself calculated at the SMD(thf):ONIOM(M06-2X/6-311+G(d):PM6) level. Selected bond distances are in Å.

Table S1. Cartesian coordinates (*x y z*) of all the compounds involved in each step of the calculated reaction mechanisms of Figure 4 in the manuscript and Figure S37 herein.

Pyridine

E(scf) = -248.241411231 a.u.

N	0.000858	-1.412695	-0.000064
C	1.141685	-0.719154	0.000111
H	2.057281	-1.303246	-0.000128
C	1.194874	0.670807	-0.000033
H	2.151650	1.179302	-0.000155
C	-0.000995	1.379438	0.000024
H	-0.001521	2.464006	-0.000025
C	-1.195731	0.669399	0.000002
H	-2.153345	1.176311	-0.000082
C	-1.140574	-0.720780	0.000061
H	-2.055631	-1.305771	-0.000159

$CAAC^{Me}$

E(scf) = -211.339209585482 a.u.

N	-0.625303	-0.119726	-0.000531
C	-1.309677	-0.181052	-1.110441
C	-1.367012	-0.208102	1.340846
C	-0.643054	-1.133086	2.300770
H	-1.214742	-1.271185	3.228904
H	0.343270	-0.749640	2.593411
H	-0.484873	-2.137988	1.877661
C	-1.512644	1.180881	1.940224
H	-2.049869	1.869747	1.266307
H	-0.541225	1.644627	2.176374
H	-2.081976	1.154890	2.878929
C	-2.704452	-0.779968	0.839358
H	-3.549341	-0.424580	1.428221
H	-2.689084	-1.868913	0.913413
C	-2.774154	-0.356399	-0.661969
C	-3.462306	1.000477	-0.834065
H	-4.533962	0.946513	-0.611193
H	-3.368026	1.375911	-1.860210
H	-3.033393	1.763974	-0.166835
C	-3.463669	-1.409713	-1.519423
H	-4.491594	-1.601038	-1.190403
H	-2.933054	-2.369432	-1.498822
H	-3.515629	-1.102128	-2.571689
C	0.817631	0.069964	-0.073978
C	1.360443	1.371006	-0.166023
C	0.502199	2.620281	-0.280687
H	-0.582442	2.336008	-0.308314
C	0.808032	3.355230	-1.591298
H	0.127243	4.202131	-1.739625
H	0.693494	2.694067	-2.459418
H	1.828363	3.754255	-1.623473
C	0.720010	3.530598	0.932782
H	0.063407	4.408084	0.897615
H	1.748845	3.902210	1.000361
H	0.502734	2.999452	1.872729
C	2.756332	1.518072	-0.191286
H	3.190321	2.518835	-0.239371

C	3.594933	0.406249	-0.169116
H	4.678274	0.537508	-0.186618
C	3.048536	-0.876852	-0.143738
H	3.713254	-1.743066	-0.164338
C	1.661181	-1.067653	-0.100879
C	1.109719	-2.483796	-0.147025
H	0.044768	-2.485040	0.215609
C	1.890359	-3.434379	0.766781
H	1.976459	-3.040540	1.786588
H	2.909243	-3.622758	0.406540
H	1.395168	-4.410609	0.838241
C	1.116096	-2.975724	-1.598602
H	0.682600	-3.979400	-1.682861
H	2.128262	-3.024154	-2.016896
H	0.529610	-2.315871	-2.250502

$LiCH_2{}^t$Bu + 1 THF molecule
E(scf) = -281.046264614791 a.u.

C	-2.062664	-0.258135	1.324262
H	-2.463322	-1.219435	1.699282
H	-2.466817	0.493969	2.022937
C	-2.700324	-0.001724	-0.058558
C	-2.269946	-1.085343	-1.051192
H	-2.724403	-0.943470	-2.035131
H	-2.561401	-2.083975	-0.691985
H	-1.182351	-1.090753	-1.184298
C	-4.242878	0.003445	0.003112
H	-4.698719	0.193263	-0.975296
H	-4.602282	0.780507	0.694281
H	-4.616151	-0.960891	0.366304
C	-2.248129	1.363265	-0.601154
H	-2.707602	1.592326	-1.571120
H	-1.159305	1.389437	-0.734014
H	-2.519175	2.168864	0.093465
C	2.484377	-1.223276	0.121685
H	1.813658	-1.693983	-0.617863
H	2.661204	-1.905190	0.972831
C	3.765870	-0.667134	-0.503192
H	3.860461	-0.968689	-1.566087
H	4.667019	-1.063449	0.000436
C	3.679835	0.864560	-0.368284
H	4.493548	1.250146	0.274819
H	3.806152	1.364550	-1.346993

LiCCPh + 3 THF molecules
E(scf) = -544.614969650038 a.u.

C	1.034817	-0.204263	0.082439
C	2.268379	-0.162348	0.069256
C	3.703054	-0.107919	0.050500
C	4.472198	-1.283941	0.049062
H	3.966221	-2.243114	0.063496
C	5.860589	-1.225947	0.029734
H	6.435436	-2.146097	0.028946
C	5.764522	1.178451	0.013336
H	6.264092	2.141424	-0.000973
C	6.514446	0.004123	0.011948
H	7.597864	0.047180	-0.003024
C	4.376000	1.125204	0.032580
H	3.795279	2.041184	0.033468
Li	-1.094727	-0.189854	0.050442
O	-2.071252	-1.816505	0.145973
C	-1.866746	-2.592925	1.346479
C	-1.971509	-2.665231	-1.018198
C	-2.175271	-4.051775	0.989467
H	-0.821119	-2.450607	1.675219
H	-2.563809	-2.150721	2.078802
C	-2.249828	-4.097517	-0.547189
H	-2.728229	-2.262810	-1.713419
H	-0.958083	-2.549841	-1.443224
H	-3.124601	-4.387607	1.443709
H	-1.396918	-4.733064	1.377874

C	2.305362	1.156029	0.239883
H	2.339563	1.789091	1.149270
H	1.588049	1.576045	-0.494587
Li	0.017783	-0.248586	1.244424
O	1.768688	-0.104092	0.696388

1

E(scf) = -238.036406192780 a.u.

N	0.576315	-0.110202	0.188039
C	1.330336	-0.162402	-0.867656
C	1.275081	-0.211441	1.543800
C	1.377907	1.172765	2.150127
H	1.961450	1.157087	3.083586
H	0.397525	1.596794	2.411922
H	1.882274	1.895216	1.484628
C	0.522888	-1.155265	2.453217
H	1.054751	-1.295374	3.407434
H	0.396386	-2.161221	2.021597
H	-0.481499	-0.790258	2.712572
C	2.637809	-0.771971	1.083370

H	-3.240413	-4.449618	-0.886665
H	-1.516037	-4.808509	-0.967478
O	-1.633877	0.484358	-1.660788
C	-2.861983	1.205103	-1.890978
C	-0.542400	1.071132	-2.404198
C	-2.629841	2.156275	-3.068470
H	-3.612692	0.420692	-2.090070
H	-3.110030	1.735394	-0.945337
C	-1.164565	1.954253	-3.489607
H	0.079239	1.644540	-1.692920
H	0.022280	0.206898	-2.796617
H	-2.824069	3.206101	-2.782125
H	-3.320188	1.944821	-3.904958
H	-0.630873	2.916909	-3.586025
H	-1.098169	1.476782	-4.484366
O	-1.696032	0.766728	1.581805
C	-2.951693	1.472312	1.655778
C	-0.645458	1.514865	2.233252
C	-2.798635	2.577933	2.704327
H	-3.166140	1.865981	0.637699
H	-3.690373	0.697218	1.923744
C	-1.326715	2.536207	3.149104
H	-0.054999	0.751645	2.771231
H	-0.031554	1.990383	1.447147
H	-3.478972	2.422295	3.561196
H	-3.066029	3.566645	2.289116
H	-1.240209	2.244906	4.212020
H	-0.849599	3.529933	3.074544

$TS_{1\to1'}$

E(scf) = -238.005926467462 a.u.

ν_{min} = -471.3 cm^{-1}

N	0.532735	-0.231163	0.144251
C	1.347999	-0.078707	-0.917640
C	1.236062	-0.416661	1.457293
C	1.533960	0.923430	2.114857
H	2.061440	0.793100	3.069663
H	0.617319	1.494823	2.342868
H	2.174789	1.569506	1.492588
C	0.432265	-1.298989	2.391308
H	0.993166	-1.520826	3.309083
H	0.171818	-2.268328	1.936680
H	-0.510577	-0.832298	2.706916

H	2.621772	-1.860328	1.150266	C	2.522752	-1.104995	0.953880
H	3.452119	-0.418508	1.712574	H	2.346768	-2.179994	0.891445
C	2.788558	-0.346682	-0.403241	H	3.362732	-0.949040	1.629668
C	3.469316	1.007659	-0.552016	C	2.772462	-0.528158	-0.468798
H	4.528835	0.960951	-0.263787	C	3.702982	0.674890	-0.435485
H	3.006839	1.782953	0.078935	H	4.713673	0.395417	-0.105748
H	3.449272	1.375911	-1.586680	H	3.353740	1.454032	0.262328
C	3.475263	-1.405122	-1.243964	H	3.824396	1.143570	-1.420777
H	4.473366	-1.648875	-0.852432	C	3.288863	-1.587317	-1.426555
H	3.626270	-1.082453	-2.283444	H	4.219489	-2.044226	-1.062251
H	2.913537	-2.347947	-1.280506	H	3.516036	-1.179785	-2.420560
C	-0.861427	0.079044	0.047684	H	2.573157	-2.406744	-1.574695
C	-1.395690	1.384864	-0.030865	C	-0.874762	0.098691	0.015307
C	-0.527160	2.626481	-0.151895	C	-1.312451	1.442527	-0.055314
H	0.542854	2.376600	0.071954	C	-0.372079	2.634905	-0.143935
C	-0.588953	3.119469	-1.602393	H	0.693973	2.274032	-0.304932
H	0.116805	3.937701	-1.781798	C	-0.716279	3.505480	-1.359669
H	-1.583788	3.479648	-1.885484	H	0.057311	4.262483	-1.537477
H	-0.328703	2.295919	-2.306678	H	-1.663522	4.044942	-1.235983
C	-0.955507	3.714701	0.835689	H	-0.810032	2.914438	-2.278840
H	-0.255340	4.559499	0.822751	C	-0.422749	3.455578	1.146256
H	-0.991365	3.340095	1.865967	H	0.313760	4.267938	1.136160
H	-1.947747	4.123647	0.607562	H	-0.210062	2.832517	2.031235
C	-2.789940	1.531607	-0.085172	H	-1.404996	3.915265	1.309473
H	-3.224019	2.533458	-0.127781	C	-2.692679	1.696228	-0.084355
C	-3.628152	0.418467	-0.103386	H	-3.049094	2.728555	-0.103335
H	-4.711877	0.551186	-0.140248	C	-3.616642	0.653576	-0.103636
C	-3.083752	-0.865549	-0.098525	H	-4.686231	0.870722	-0.122538
H	-3.748500	-1.730864	-0.156137	C	-3.172385	-0.667542	-0.121581
C	-1.697627	-1.060076	-0.030674	H	-3.901063	-1.478840	-0.184958
C	-1.145703	-2.475206	-0.102648	C	-1.804303	-0.967362	-0.067896
H	-0.081617	-2.488875	0.257302	C	-1.360142	-2.419778	-0.169105
C	-1.159764	-2.937265	-1.562685	H	-0.303724	-2.510967	0.198485
H	-0.695067	-3.922463	-1.679489	C	-1.390281	-2.851338	-1.642395
H	-0.594687	-2.230057	-2.214866	H	-2.236128	-3.563208	-1.854491
H	-2.173730	-3.004694	-1.972168	H	-0.430279	-3.372875	-1.923225
C	-1.929025	-3.438244	0.795098	H	-1.509519	-1.979460	-2.338209
H	-1.439067	-4.418710	0.846519	C	-2.214140	-3.338799	0.711811
H	-2.949703	-3.615697	0.433326	H	-1.816403	-4.359110	0.714489
H	-2.011961	-3.064194	1.822677	H	-3.254799	-3.404376	0.372708
B	0.816428	-0.058838	-2.371316	H	-2.234718	-2.994587	1.753939
H	0.159146	-1.089151	-2.550287	B	0.976396	0.441993	-2.293897

H	1.756057	-0.004642	-3.135032
H	0.07632	0.918237	-2.477635

1′

E(scf) = -238.016012909970 a.u.

N	-0.551191	0.117974	0.182270
C	-1.396863	-0.468045	-0.854631
H	-1.404684	-1.575080	-0.812637
C	-1.208719	-0.044515	1.491346
C	-1.156687	-1.470372	2.060221
H	-1.715090	-1.549121	2.999779
H	-0.126614	-1.786790	2.279866
H	-1.584782	-2.210440	1.375016
C	-0.631277	0.922251	2.519205
H	-1.235230	0.948189	3.433334
H	-0.586964	1.951805	2.128527
H	0.390328	0.654935	2.814654
C	-2.669847	0.328651	1.113692
H	-3.396103	-0.230270	1.706749
H	-2.843796	1.385534	1.321500
C	-2.833948	0.041998	-0.419079
C	-3.881174	-1.028503	-0.697843
H	-4.884350	-0.704842	-0.390792
H	-3.677111	-1.963817	-0.161977
H	-3.945659	-1.278789	-1.763904
C	-3.195825	1.324956	-1.168697
H	-4.155476	1.735024	-0.825604
H	-3.312346	1.175057	-2.252235
H	-2.452495	2.119793	-1.016953
C	0.882576	0.016303	0.014344
C	1.591226	-1.196387	-0.154338
C	0.927723	-2.562211	-0.219825
H	-0.190164	-2.448838	-0.144019
C	1.385328	-3.440413	0.950247
H	0.871937	-4.408781	0.949552
H	1.168802	-2.957550	1.915332
H	2.461122	-3.647213	0.925529
C	1.214295	-3.234475	-1.568524
H	0.657881	-4.173467	-1.671428
H	2.275273	-3.475293	-1.701311
H	0.922279	-2.591283	-2.407857
C	2.989235	-1.159082	-0.295264

H	1.802771	0.372156	-3.155426
H	1.456225	1.481902	-1.306627
H	-0.173253	0.683493	-2.534008

A1

E(scf) = -449.409137784521 a.u.

N	-4.762995	-0.411763	0.544955
N	5.770982	0.331717	-0.000108
C	-4.565058	-1.603829	-0.377795
H	-4.823643	-1.402431	-1.416227
C	-5.515692	-0.837729	1.808873
C	-4.872892	-0.218428	3.035810
H	-5.180649	-0.737951	3.954517
H	-5.151391	0.836419	3.171143
H	-3.770320	-0.251557	3.011302
C	-6.975968	-0.425307	1.722173
H	-7.499980	-0.902570	0.874727
H	-7.110016	0.660128	1.621692
H	-7.523880	-0.718299	2.629605
C	-5.352629	-2.375392	1.789892
H	-6.183395	-2.858565	2.303981
H	-4.447282	-2.651881	2.327653
C	-5.238547	-2.815554	0.300560
C	-6.610656	-3.110846	-0.298651
H	-7.120651	-3.926410	0.227770
H	-6.545039	-3.408985	-1.352209
H	-7.278288	-2.231082	-0.251129
C	-4.345663	-4.043344	0.166808
H	-4.763403	-4.912447	0.689445
H	-3.340725	-3.882468	0.576739
H	-4.209975	-4.335532	-0.882440
C	-4.988947	0.917331	-0.022208
C	-6.011819	1.172667	-0.968912
C	-7.008675	0.136382	-1.462873
H	-7.022002	-0.747829	-0.759485
C	-6.592892	-0.361499	-2.850561
H	-7.301841	-1.101445	-3.241323
H	-5.602704	-0.844129	-2.822479
H	-6.535876	0.448918	-3.586496
C	-8.439409	0.688064	-1.477296
H	-9.167065	-0.108312	-1.676983
H	-8.588717	1.453106	-2.249479
H	-8.711541	1.141678	-0.516537

H	3.541357	-2.093987	-0.407917
C	3.681968	0.047053	-0.298819
H	4.766859	0.057571	-0.402638
C	2.977953	1.246213	-0.183803
H	3.523351	2.190985	-0.219944
C	1.586875	1.252313	-0.034005
C	0.860603	2.588428	0.002663
H	-0.149015	2.444966	0.480199
C	1.603842	3.635109	0.840411
H	1.002803	4.544007	0.961840
H	2.554427	3.940791	0.387516
H	1.830850	3.261120	1.845733
C	0.643521	3.074899	-1.434788
H	0.039568	3.987272	-1.465338
H	0.105703	2.304890	-2.035413
H	1.584911	3.286335	-1.951058
B	-1.21414	0.082011	-2.292605
H	-0.58314	1.092124	-2.47469
H	-1.750216	-0.465731	-3.207366

C	-6.126403	2.459255	-1.520446
H	-6.899173	2.656062	-2.268213
C	-5.276579	3.490762	-1.134519
H	-5.383506	4.485645	-1.570794
C	-4.286474	3.242180	-0.188041
H	-3.620313	4.054849	0.112758
C	-4.121395	1.969023	0.378529
C	-2.971636	1.814630	1.365308
H	-3.033400	0.826954	1.898767
C	-3.018428	2.899301	2.448137
H	-4.001219	2.952109	2.931090
H	-2.797619	3.898919	2.054742
H	-2.280849	2.700975	3.235876
C	-1.643134	1.844611	0.598639
H	-0.791641	1.683588	1.269527
H	-1.474548	2.792588	0.078373
H	-1.621943	1.038482	-0.160742
C	5.607326	0.831316	1.194527
C	6.870276	0.906477	-0.904605
C	7.633173	-0.202450	-1.603494
H	8.481595	0.196640	-2.176521
H	7.009674	-0.760636	-2.314288
H	8.050208	-0.937282	-0.895732
C	6.256727	1.857760	-1.919052
H	5.710369	2.685483	-1.435432
H	5.551001	1.355416	-2.600314
H	7.026115	2.318436	-2.552859
C	7.701561	1.638036	0.163576
H	8.172380	2.538569	-0.228787
H	8.496403	0.981599	0.522904
C	6.691582	1.920073	1.320340
C	5.995617	3.272855	1.147394
H	5.177328	3.402770	1.865970
H	5.563713	3.385113	0.140820
H	6.686922	4.110020	1.295233
C	7.358302	1.841177	2.687843
H	8.180879	2.558592	2.787518
H	7.772529	0.844539	2.885479
H	6.646331	2.051586	3.495857
C	4.875478	-0.728468	-0.445641
C	3.632664	-0.407551	-1.035106
C	3.122369	1.018537	-1.163037

H	3.855999	1.728204	-0.698138
C	2.968133	1.400043	-2.639214
H	2.656549	2.445392	-2.751467
H	2.221404	0.786280	-3.155838
H	3.919253	1.283425	-3.181890
C	1.799386	1.178123	-0.403552
H	1.898371	0.867980	0.644221
H	0.990169	0.582127	-0.841194
H	1.468055	2.223379	-0.402205
C	2.811355	-1.452920	-1.486783
H	1.856730	-1.219952	-1.963086
C	3.189743	-2.782975	-1.321683
H	2.540752	-3.584397	-1.679111
C	4.389816	-3.091471	-0.680576
H	4.658392	-4.138219	-0.523251
C	5.246295	-2.078801	-0.229711
C	6.507294	-2.457530	0.530749
H	7.237225	-1.601480	0.495812
C	7.215744	-3.667633	-0.086653
H	7.397381	-3.530501	-1.159299
H	6.641940	-4.595423	0.028081
H	8.190585	-3.838796	0.386764
C	6.145930	-2.713126	1.998080
H	7.035258	-2.950981	2.593677
H	5.445758	-3.548616	2.114559
H	5.676434	-1.832759	2.455922
B	-3.011046	-1.585856	-0.182001
H	-2.321431	-1.093613	-1.042943
H	-2.500718	-2.365757	0.575009

TS$_{A1\rightarrow 4}$

E(scf) = -449.404092368073 a.u.

ν_{min} = -119.0 cm^{-1}

N	2.588159	-0.535252	-0.620269
N	-2.688572	-0.462623	0.509753
C	1.390102	-1.342645	-0.295697
H	1.540467	-1.858942	0.678624
C	3.653532	-1.383349	-1.160427
C	4.711818	-0.564939	-1.896237
H	5.407844	-1.208236	-2.445664
H	5.308442	0.052588	-1.215445
H	4.256438	0.120505	-2.629141
C	4.354695	-2.282656	-0.125887

4

E(scf) = -449.440175002476 a.u.

N	-2.429560	-0.492108	0.684789
N	2.460637	-0.509190	-0.633479
C	-1.332412	-1.401250	0.279615
H	-1.615533	-1.913303	-0.671186
C	-3.524944	-1.229114	1.312016
C	-4.451733	-0.310222	2.106431
H	-5.178531	-0.882729	2.693272
H	-5.019812	0.367303	1.459099
H	-3.891681	0.320385	2.812906
C	-4.381225	-2.074854	0.350037
H	-3.778576	-2.801904	-0.208752

H	3.647355	-2.939102	0.395973
H	4.885840	-1.692629	0.635755
H	5.098958	-2.931847	-0.598298
C	2.815094	-2.239331	-2.135908
H	3.301881	-3.182213	-2.396626
H	2.686420	-1.676969	-3.063547
C	1.429575	-2.440171	-1.430956
C	1.363801	-3.840913	-0.815603
H	1.446353	-4.625412	-1.575641
H	0.409164	-4.009056	-0.289281
H	2.171798	-4.009713	-0.091600
C	0.295949	-2.278105	-2.440190
H	0.388294	-2.980861	-3.275268
H	0.274576	-1.265454	-2.868954
H	-0.683567	-2.450662	-1.979102
C	2.839053	0.650955	0.165961
C	3.039153	0.653129	1.567628
C	2.998496	-0.602616	2.422380
H	2.669287	-1.475666	1.791077
C	1.973539	-0.465837	3.555717
H	1.860397	-1.407232	4.105134
H	0.984367	-0.192916	3.166753
H	2.254417	0.300837	4.286373
C	4.393311	-0.917213	2.976536
H	4.387403	-1.826304	3.588376
H	4.787919	-0.109416	3.602030
H	5.114510	-1.080164	2.158782
C	3.276235	1.871998	2.224796
H	3.446839	1.875839	3.302346
C	3.290874	3.075621	1.527505
H	3.476254	4.012143	2.051609
C	3.053494	3.078753	0.152485
H	3.037113	4.029898	-0.381953
C	2.827219	1.886394	-0.543683
C	2.519822	1.956556	-2.032122
H	2.741886	0.954571	-2.496553
C	3.397161	2.985140	-2.757630
H	4.460998	2.834605	-2.541045
H	3.152811	4.016744	-2.479120
H	3.275256	2.911969	-3.844564
C	1.030232	2.254451	-2.237573
H	0.776262	2.337400	-3.298090
H	-4.914093	-1.448610	-0.380670
H	-5.142672	-2.646009	0.890648
C	-2.699014	-2.145652	2.240118
H	-3.249667	-3.032588	2.564181
H	-2.441382	-1.579750	3.138520
C	-1.402637	-2.491751	1.430174
C	-1.550039	-3.891940	0.825261
H	-1.643229	-4.662606	1.598000
H	-0.679994	-4.166281	0.207050
H	-2.441333	-3.968229	0.186893
C	-0.187975	-2.460500	2.353518
H	-0.307571	-3.135436	3.208066
H	-0.012600	-1.451625	2.759865
H	0.727766	-2.759513	1.832952
C	-2.627383	0.733491	-0.051129
C	-2.980833	0.794484	-1.420960
C	-3.139121	-0.432739	-2.301540
H	-2.823172	-1.347267	-1.726572
C	-2.231272	-0.344045	-3.535906
H	-2.340794	-1.228650	-4.173946
H	-1.171302	-0.278595	-3.248252
H	-2.452592	0.528958	-4.159826
C	-4.606192	-0.610835	-2.711615
H	-4.745453	-1.500933	-3.335263
H	-4.989498	0.244450	-3.278293
H	-5.250085	-0.730934	-1.824602
C	-3.185680	2.048098	-2.021475
H	-3.483614	2.097464	-3.069956
C	-3.008286	3.228168	-1.306749
H	-3.170585	4.191962	-1.786667
C	-2.606785	3.169566	0.028633
H	-2.438672	4.099222	0.574430
C	-2.418057	1.942411	0.673064
C	-1.968663	1.945760	2.127007
H	-2.196868	0.942175	2.581485
C	-2.723771	2.992582	2.958007
H	-3.808708	2.908298	2.827960
H	-2.445309	4.019021	2.693038
H	-2.515944	2.872732	4.027589
C	-0.454561	2.164871	2.207273
H	-0.105560	2.194766	3.243409
H	-0.140308	3.092779	1.721610

H	0.718303	3.180556	-1.747176
H	0.402551	1.432467	-1.815205
C	-1.598934	-1.158808	0.552011
C	-3.932905	-1.085320	1.163666
C	-4.695461	-0.051152	1.961812
H	-5.578543	-0.495550	2.445749
H	-5.067496	0.775784	1.340740
H	-4.096387	0.394780	2.769998
C	-4.807208	-1.695114	0.085674
H	-4.271069	-2.441864	-0.524877
H	-5.218272	-0.939536	-0.607329
H	-5.673194	-2.215813	0.519177
C	-3.241175	-2.145288	2.042446
H	-3.862514	-3.028625	2.177359
H	-3.043591	-1.725546	3.029732
C	-1.891305	-2.452190	1.325670
C	-2.034967	-3.587406	0.318558
H	-1.089393	-3.772645	-0.224601
H	-2.812960	-3.385055	-0.433624
H	-2.303117	-4.532955	0.806136
C	-0.764911	-2.725273	2.306186
H	-0.936017	-3.641764	2.884423
H	-0.635869	-1.908747	3.029263
H	0.201363	-2.844526	1.792531
C	-2.696822	0.847310	-0.133563
C	-2.938979	0.957446	-1.521627
C	-3.139307	-0.240550	-2.436066
H	-2.949673	-1.188456	-1.868635
C	-4.583587	-0.271013	-2.949826
H	-4.767109	-1.153505	-3.574523
H	-4.831690	0.608003	-3.555750
H	-5.305109	-0.306419	-2.116933
C	-2.145364	-0.200754	-3.602429
H	-1.104872	-0.128610	-3.248138
H	-2.313601	0.650333	-4.272598
H	-2.215022	-1.108109	-4.214420
C	-2.978486	2.238061	-2.094831
H	-3.176806	2.342087	-3.164046
C	-2.761022	3.378360	-1.325079
H	-2.799115	4.366694	-1.787328
C	-2.478289	3.254762	0.034299
H	-2.279375	4.151948	0.624845

H	0.087578	1.329897	1.701677
C	1.306777	-1.118722	-0.660650
C	3.592876	-1.135852	-1.444643
C	4.374689	-0.078121	-2.190596
H	5.182431	-0.530085	-2.787233
H	4.856988	0.648320	-1.521146
H	3.757628	0.495104	-2.899659
C	4.497999	-1.922327	-0.518018
H	3.955444	-2.685360	0.067189
H	5.034885	-1.278841	0.202018
H	5.274108	-2.462096	-1.080609
C	2.758837	-2.036870	-2.375515
H	3.304124	-2.929676	-2.675041
H	2.506034	-1.483633	-3.281153
C	1.459212	-2.353430	-1.583238
C	1.630125	-3.587093	-0.709247
H	0.731888	-3.790578	-0.097021
H	2.485042	-3.504598	-0.019153
H	1.799500	-4.489179	-1.312214
C	0.253246	-2.478821	-2.491385
H	0.367732	-3.294559	-3.216191
H	0.063704	-1.558497	-3.067507
H	-0.668961	-2.683769	-1.919551
C	2.609236	0.739951	0.105181
C	2.994200	0.725357	1.464218
C	3.211333	-0.549997	2.261703
H	2.912911	-1.439171	1.648787
C	4.694650	-0.695621	2.621487
H	4.883936	-1.627623	3.167869
H	5.055901	0.124560	3.252446
H	5.325919	-0.714937	1.717956
C	2.337374	-0.551030	3.521184
H	1.268035	-0.447324	3.271129
H	2.586713	0.262773	4.211641
H	2.446639	-1.488438	4.079914
C	3.169090	1.951636	2.125218
H	3.482300	1.956969	3.171996
C	2.940501	3.160354	1.472569
H	3.079142	4.104443	2.003183
C	2.521313	3.162421	0.142805
H	2.318507	4.114049	-0.354150
C	2.351984	1.963417	-0.563120

C	-2.433382	1.996468	0.651134
C	-2.053055	1.929041	2.122882
H	-2.249311	0.898592	2.521120
C	-2.885939	2.906483	2.960860
H	-3.960635	2.796962	2.773831
H	-2.630296	3.954088	2.759001
H	-2.724552	2.744335	4.033675
C	-0.555221	2.211271	2.268341
H	-0.219360	2.099299	3.303547
H	-0.281292	3.219160	1.940541
H	0.044282	1.499107	1.644503
B	0.168200	-0.305336	-0.188936
H	0.312815	0.517735	0.711429
H	-0.208190	0.203954	-1.234343

A2

E(scf) = -486.295261078228 a.u.

N	0.811908	0.064282	0.099197
N	-6.110961	-0.087797	-1.066169
C	0.090653	-0.586367	-0.991425
H	0.126468	-1.691144	-0.924960
C	0.073360	-0.124907	1.361442
C	0.271923	-1.504675	2.005516
H	-0.386888	-1.647969	2.868950
H	1.298363	-1.639579	2.367727
H	0.067083	-2.324023	1.304840
C	0.442412	0.953317	2.375062
H	-0.229608	0.942667	3.240308
H	0.391966	1.960948	1.931085
H	1.463044	0.832140	2.757647
C	-1.398984	0.032995	0.870203
H	-2.054133	-0.701408	1.342319
H	-1.790804	1.013692	1.145559
C	-1.395446	-0.141256	-0.686946
C	-2.380175	-1.207966	-1.147987
H	-2.202879	-2.179101	-0.670091
H	-2.336372	-1.374439	-2.231483
H	-3.416079	-0.927137	-0.917558
C	-1.703978	1.184860	-1.383081
H	-2.705646	1.553644	-1.124816
H	-1.691405	1.108184	-2.480363
H	-0.997385	1.975710	-1.096664
C	2.258475	0.043777	0.054989

C	1.862311	2.038598	-2.001692
H	1.997511	1.044078	-2.504261
C	2.663906	3.063937	-2.812504
H	3.745150	2.908037	-2.719344
H	2.460298	4.096064	-2.501092
H	2.418160	3.003320	-3.879946
C	0.368041	2.370451	-2.010183
H	-0.036697	2.392792	-3.026748
H	0.147501	3.336913	-1.545679
H	-0.208097	1.598831	-1.439597
B	0.006133	-0.512804	0.041993
H	-0.267319	0.364085	-0.807579
H	0.361813	0.088082	1.060824

$TS_{A2\rightarrow 5}$

E(scf) = -486.286484618782 a.u.

ν_{min} = -138.9 cm^{-1}

N	-0.860680	-0.323605	0.724763
N	3.062056	0.175855	-0.864825
C	0.599446	-0.163758	0.647968
H	0.922337	0.745558	1.197974
C	-1.304519	-0.666197	2.084434
C	-1.732509	0.551751	2.917429
H	-1.866110	0.296796	3.973493
H	-2.683966	0.969162	2.568398
H	-0.991432	1.362239	2.863965
C	-2.454627	-1.671385	2.057405
H	-2.725374	-2.001306	3.066360
H	-2.197022	-2.568363	1.476804
H	-3.358839	-1.251223	1.601430
C	-0.000176	-1.293223	2.682814
H	0.400327	-0.643195	3.465116
H	-0.187798	-2.260448	3.153379
C	1.031822	-1.380135	1.512960
C	2.454409	-1.238799	2.055577
H	2.637636	-0.255131	2.501497
H	3.207083	-1.383434	1.273531
H	2.663137	-1.984586	2.832250
C	0.886674	-2.699972	0.754714
H	1.055969	-3.566154	1.401895
H	1.592938	-2.774337	-0.078016
H	-0.128552	-2.801453	0.329885

C	3.043590	-1.129908	-0.040335
C	2.458527	-2.525213	-0.183463
H	1.339830	-2.487003	-0.054338
C	3.006895	-3.475655	0.886875
H	2.487463	-4.440859	0.865790
H	2.884563	-3.062935	1.895788
H	4.074954	-3.685450	0.754557
C	2.733913	-3.058540	-1.595696
H	2.251123	-4.029302	-1.756994
H	3.803813	-3.195703	-1.789609
H	2.353331	-2.374841	-2.365222
C	4.444301	-1.018034	-0.050489
H	5.053698	-1.921633	-0.110641
C	5.068799	0.223704	0.009849
H	6.156537	0.292098	0.012210
C	4.294307	1.383427	0.047336
H	4.789573	2.356103	0.056584
C	2.896604	1.315520	0.059207
C	2.100646	2.610215	-0.004570
H	1.057445	2.420371	0.375710
C	2.700188	3.713994	0.873717
H	2.043318	4.591109	0.909767
H	3.673993	4.061023	0.508041
H	2.844601	3.375346	1.906277
C	2.000304	3.054297	-1.468940
H	1.365107	3.938626	-1.580413
H	1.553577	2.247847	-2.095557
H	2.976906	3.294189	-1.900557
C	-6.091744	-1.085505	-0.179450
H	-6.575056	-2.008764	-0.485968
C	-5.497859	-0.986436	1.075247
H	-5.515337	-1.831740	1.753210
C	-4.893436	0.214125	1.429198
H	-4.423144	0.332865	2.399592
C	-4.904626	1.260295	0.514001
H	-4.448542	2.216487	0.743435
C	-5.524922	1.059528	-0.715384
H	-5.554547	1.857553	-1.451863
B	0.376230	-0.052607	-2.418504
H	-0.064783	-0.630017	-3.365583
H	0.985699	0.976527	-2.564648

C	-1.706679	0.321193	-0.247201
C	-1.881823	1.724037	-0.328209
C	-1.070031	2.695128	0.511364
H	-0.571697	2.138388	1.353222
C	-1.942539	3.781529	1.149102
H	-1.357487	4.412034	1.828882
H	-2.763663	3.349274	1.733224
H	-2.394665	4.448585	0.405615
C	0.027475	3.302315	-0.372707
H	0.772261	3.843312	0.217515
H	-0.372633	4.001755	-1.113675
H	0.556089	2.502269	-0.939504
C	-2.777169	2.248707	-1.272777
H	-2.926664	3.327947	-1.330496
C	-3.472149	1.414165	-2.144861
H	-4.174051	1.835409	-2.863945
C	-3.249124	0.037734	-2.109873
H	-3.765992	-0.606150	-2.822802
C	-2.363982	-0.524893	-1.181757
C	-2.081994	-2.017154	-1.248297
H	-1.547938	-2.339079	-0.302168
C	-3.369699	-2.840755	-1.349381
H	-3.164630	-3.912921	-1.247822
H	-3.880820	-2.704117	-2.309393
H	-4.083752	-2.572791	-0.561887
C	-1.135717	-2.305435	-2.420123
H	-0.919976	-3.374181	-2.512950
H	-0.160167	-1.785566	-2.269026
H	-1.537116	-1.966857	-3.379686
C	3.598122	1.266150	-0.312991
H	2.900181	1.989712	0.094717
C	4.970231	1.466781	-0.266063
H	5.370012	2.364671	0.188428
C	5.803412	0.498839	-0.816253
H	6.879843	0.627005	-0.798467
C	5.237339	-0.632439	-1.391799
H	5.849679	-1.408682	-1.833621
C	3.854300	-0.756661	-1.395596
H	3.358545	-1.615995	-1.832821
B	1.002060	-0.030680	-0.890218
H	0.750372	1.049265	-1.388388
H	0.901037	-1.004897	-1.606684

2a (with 1 THF molecule)
E(scf) = -519.165387781066 a.u.

C	-1.427110	1.313646	0.347105
H	-1.583311	1.560549	1.420060
C	-3.530040	0.073392	0.145078
C	-4.076399	-0.353259	1.517225
H	-5.129266	-0.079636	1.638420
H	-3.517913	0.115161	2.341391
H	-4.010521	-1.436738	1.664784
C	-4.206837	-0.778615	-0.929318
H	-5.294355	-0.649538	-0.920709
H	-4.007094	-1.847792	-0.794711
H	-3.852300	-0.513889	-1.936574
C	-3.787405	1.601875	-0.082913
H	-4.171693	2.055319	0.834852
H	-4.543439	1.778874	-0.851362
C	-2.403914	2.243655	-0.431845
C	-2.160797	2.200791	-1.942548
H	-2.888820	2.804544	-2.492582
H	-2.234361	1.168374	-2.323334
H	-1.165484	2.571022	-2.205439
C	-2.366955	3.691775	0.060132
H	-3.198467	4.278119	-0.346002
H	-1.441165	4.196072	-0.244971
H	-2.424720	3.764722	1.151375
C	-1.295948	-1.188992	0.156350
C	-0.931542	-1.844065	-1.054791
C	-1.311419	-1.287353	-2.417186
H	-2.098865	-0.481678	-2.284558
C	-1.903493	-2.365397	-3.328684
H	-2.308549	-1.928854	-4.249628
H	-2.725404	-2.901771	-2.838991
H	-1.163943	-3.115966	-3.630246
C	-0.090535	-0.614388	-3.058185
H	-0.334943	-0.203465	-4.044978
H	0.749767	-1.300091	-3.198071
H	0.253391	0.244395	-2.438638
C	-0.141279	-2.999822	-1.001310
H	0.126255	-3.514957	-1.926648
C	0.311472	-3.504325	0.217706
H	0.922925	-4.406788	0.243324
C	-0.017097	-2.846469	1.400759

2c (with 3 THF molecules)
E(scf) = -782.708181968173 a.u.

H	-0.314938	0.133558	-1.153244
H	-0.335863	-0.161462	0.837835
N	-2.620414	1.587581	-0.079341
C	-1.208958	1.928264	0.163545
H	-1.083260	2.297724	1.204884
C	-3.477699	2.780798	-0.047028
C	-4.066560	3.085052	1.341218
H	-4.540952	4.070748	1.374644
H	-3.296955	3.064069	2.124834
H	-4.830212	2.353434	1.627812
C	-4.626957	2.675087	-1.050481
H	-5.221820	3.594434	-1.078886
H	-5.311262	1.853951	-0.809567
H	-4.258744	2.493236	-2.069307
C	-2.463140	3.901412	-0.454147
H	-2.330032	4.606607	0.370767
H	-2.821814	4.489997	-1.301841
C	-1.105757	3.183668	-0.748862
C	-1.007707	2.800610	-2.228014
H	-1.093340	3.672124	-2.883784
H	-1.812347	2.096261	-2.506839
H	-0.056705	2.312767	-2.460925
C	0.060196	4.095244	-0.364445
H	0.015069	5.054109	-0.892529
H	1.026043	3.640989	-0.613190
H	0.081235	4.318744	0.707594
C	-3.136165	0.278843	0.228419
C	-3.542609	-0.517260	-0.878372
C	-3.241494	-0.095254	-2.306746
H	-3.016794	1.015473	-2.325666
C	-4.420939	-0.339642	-3.252411
H	-4.238275	0.105286	-4.237658
H	-5.346410	0.103355	-2.865944
H	-4.616420	-1.405749	-3.414833
C	-1.975129	-0.820529	-2.780145
H	-1.709001	-0.543236	-3.804524
H	-2.077462	-1.908290	-2.747266
H	-1.113056	-0.539713	-2.127517
C	-4.171655	-1.745805	-0.645498
H	-4.510587	-2.348630	-1.488700

H	0.349956	-3.239215	2.351618	C	-4.365161	-2.215989	0.654486
C	-0.816610	-1.693370	1.392598	H	-4.873110	-3.164745	0.821972
C	-1.100459	-1.010170	2.719840	C	-3.885719	-1.477623	1.733467
H	-1.870790	-0.201756	2.567383	H	-3.999898	-1.870619	2.744703
C	0.175768	-0.340351	3.240525	C	-3.258279	-0.236752	1.541152
H	-0.024880	0.276514	4.123056	C	-2.669435	0.469820	2.749370
H	0.607742	0.331043	2.464597	H	-2.418835	1.532622	2.474236
H	0.949958	-1.062112	3.516624	C	-3.650644	0.528464	3.924675
C	-1.675349	-1.994354	3.744318	H	-3.253008	1.141259	4.742353
H	-2.000040	-1.475262	4.654091	H	-3.866292	-0.461603	4.342864
H	-0.948461	-2.754445	4.052925	H	-4.610293	0.968398	3.628414
H	-2.548210	-2.527268	3.348242	C	-1.363061	-0.232966	3.142240
C	0.802594	2.852985	0.164229	H	-0.813905	0.335354	3.898955
H	0.356144	3.450543	-0.647929	H	-0.701001	-0.337715	2.250905
H	0.443491	3.329775	1.090944	H	-1.530673	-1.237697	3.540273
C	2.329254	3.084514	0.106240	C	1.343291	1.198920	0.078659
C	2.941495	2.426523	-1.133074	C	2.505269	1.546288	0.194419
H	3.999047	2.691545	-1.239467	C	3.875605	1.948064	0.326556
H	2.421211	2.737989	-2.044576	C	4.230571	3.305330	0.335851
H	2.883026	1.334139	-1.079629	H	3.454473	4.056879	0.240037
C	2.601675	4.592921	0.037165	C	5.561525	3.683687	0.464426
H	3.675398	4.810891	0.027614	H	5.820110	4.737168	0.468932
H	2.163061	5.107535	0.898327	C	6.218216	1.369345	0.579451
H	2.163955	5.027430	-0.867287	H	6.989211	0.612415	0.676474
C	3.019283	2.535059	1.357684	C	6.560159	2.719823	0.586865
H	4.090274	2.765799	1.350131	H	7.597362	3.019415	0.687685
H	2.917205	1.441457	1.430022	C	4.889103	0.985716	0.450113
H	2.590230	2.964252	2.268668	H	4.616848	-0.071027	0.441796
C	3.660567	-1.867494	-1.262822	B	-0.164282	0.704751	-0.054183
H	3.353245	-1.192728	-2.082363	Li	1.322844	-1.227273	-0.117805
H	3.285171	-2.892097	-1.442360	O	0.357667	-2.877348	-0.308039
C	5.159089	-1.804061	-0.965142	C	0.462121	-3.562352	-1.575650
H	5.721719	-1.340084	-1.796231	C	-0.975867	-3.014807	0.227965
H	5.587936	-2.816981	-0.844570	C	-0.666639	-4.598220	-1.612469
C	5.289202	-0.988281	0.331004	H	0.380903	-2.805243	-2.376060
H	6.030322	-1.433578	1.020170	H	1.476623	-3.997848	-1.570363
H	5.654278	0.035866	0.123816	C	-1.570707	-4.275207	-0.409468
C	3.889664	-0.965759	0.943110	H	-0.830396	-3.085516	1.319464
H	3.764308	-1.671829	1.784733	H	-1.538485	-2.097696	-0.031081
H	3.552944	0.053892	1.241683	H	-0.274054	-5.629174	-1.550804
H	0.422352	0.809332	-1.039482	H	-1.226041	-4.551481	-2.564202
H	0.693158	0.625477	0.989375	H	-1.597215	-5.116866	0.305726

Li	1.327144	-0.623578	-0.179534
B	0.178082	1.351229	0.080479
N	-2.067333	0.018286	0.042508
O	2.967097	-1.422398	-0.073469
H	-2.619372	-4.115161	-0.719644
O	2.456186	-1.638781	1.383048
C	2.491109	-3.025316	1.792443
C	2.451719	-0.768037	2.538387
C	2.077619	-3.076878	3.264464
H	1.801862	-3.563672	1.114435
H	3.529837	-3.364220	1.625720
C	2.210711	-1.634675	3.778500
H	3.438418	-0.270559	2.542371
H	1.654333	-0.024009	2.367203
H	2.711108	-3.775404	3.840228
H	1.041695	-3.445406	3.381073
H	3.042241	-1.539502	4.500717
H	1.303389	-1.317881	4.325184
O	2.420389	-1.487093	-1.683259
C	3.793309	-1.818935	-1.378570
C	2.361614	-0.487491	-2.725888
C	4.646770	-1.351348	-2.560782
H	4.061174	-1.307907	-0.426054
H	3.787049	-2.913405	-1.234941
C	3.712142	-0.511102	-3.447672
H	1.509223	-0.796629	-3.355681
H	2.154034	0.490241	-2.254565
H	5.066242	-2.206557	-3.121004
H	5.518012	-0.761616	-2.221817
H	3.624368	-0.944920	-4.459978
H	4.105182	0.511853	-3.596095

$TS_{4\rightarrow A3}$

E(scf) = –449.399065556358 a.u.
ν_{min} = –670.3 cm^{-1}

N	2.485457	-0.682715	-0.163030
N	-2.294024	-0.184533	0.952923
C	1.406696	-1.515304	-0.029784
H	-0.650296	-2.402804	0.241198
C	3.783820	-1.388074	-0.521158
C	4.580630	-0.632901	-1.569389
H	5.495800	-1.178273	-1.839734
H	4.899193	0.360906	-1.227175
H	4.024387	-0.478358	-2.511601
C	4.641850	-1.589559	0.720138
H	4.062286	-1.965936	1.579964
H	5.140531	-0.661118	1.043837

A3

E(scf) = –449.427860905526 a.u.

N	2.221134	-0.789976	0.122915
N	-2.397010	-0.025967	0.716979
C	1.140570	-1.286322	-0.775267
H	-1.119487	-1.617607	-1.238634
C	3.351125	-1.777611	0.223112
C	4.735088	-1.142474	0.087929
H	5.527213	-1.896159	0.177747
H	4.925294	-0.391734	0.864926
H	4.892645	-0.642870	-0.882207
C	3.292809	-2.500887	1.569980
H	2.303542	-2.960203	1.747842
H	3.478408	-1.819604	2.408944

H	5.442398	-2.318797	0.538538	H	4.036574	-3.302341	1.630381
C	3.248520	-2.733016	-1.047424	C	3.083859	-2.743278	-0.953340
H	3.968799	-3.541284	-0.925823	H	3.515932	-3.733176	-0.795733
H	3.029389	-2.640679	-2.113824	H	3.538825	-2.330634	-1.859796
C	1.930483	-2.962767	-0.278955	C	1.549022	-2.773063	-1.133826
C	2.134964	-3.607860	1.087995	C	0.901578	-3.794981	-0.205232
H	2.462808	-4.650524	1.006494	H	1.266844	-4.810721	-0.393313
H	1.203772	-3.606210	1.683288	H	-0.195394	-3.828496	-0.308868
H	2.892798	-3.080807	1.691521	H	1.113676	-3.569573	0.859818
C	0.936760	-3.771060	-1.097669	C	1.169552	-3.099472	-2.576206
H	1.270508	-4.808383	-1.232446	H	1.524918	-4.095810	-2.869487
H	0.775521	-3.363107	-2.102994	H	1.593506	-2.391556	-3.299083
H	-0.050548	-3.818471	-0.603941	H	0.083512	-3.096299	-2.731048
C	2.353651	0.739655	-0.425121	C	2.589106	0.609895	0.009092
C	2.861311	1.686485	0.495753	C	2.602435	1.360951	1.214382
C	3.374174	1.339020	1.884019	C	2.189787	0.781469	2.556315
H	3.139034	0.265831	2.108081	H	1.889345	-0.298787	2.426324
C	2.690733	2.206207	2.949959	C	0.964664	1.531903	3.096432
H	2.981422	1.894628	3.960446	H	0.641834	1.133481	4.064370
H	1.592459	2.134009	2.891630	H	0.116410	1.438195	2.394700
H	2.947550	3.268001	2.858443	H	1.151565	2.601681	3.238803
C	4.894208	1.537527	1.954362	C	3.355055	0.821041	3.551017
H	5.277646	1.329020	2.960559	H	3.111392	0.279002	4.472221
H	5.195894	2.560203	1.701953	H	3.622708	1.843312	3.841943
H	5.422245	0.861903	1.260880	H	4.258161	0.359712	3.133224
C	2.915130	3.041253	0.123687	C	3.004737	2.704620	1.194109
H	3.316983	3.773927	0.827907	H	3.024559	3.273952	2.125173
C	2.479151	3.466124	-1.126028	C	3.377882	3.326909	0.005579
H	2.555638	4.516822	-1.409229	H	3.687945	4.371390	0.004346
C	1.931232	2.538826	-2.011324	C	3.349314	2.599936	-1.181581
H	1.569020	2.880600	-2.984223	H	3.638337	3.090165	-2.113405
C	1.832776	1.181487	-1.675731	C	2.969420	1.248959	-1.201699
C	1.156777	0.280482	-2.699785	C	3.026657	0.555942	-2.554912
H	0.911805	-0.730187	-2.224368	H	2.665131	-0.503706	-2.456652
C	2.059983	0.037471	-3.908763	C	4.471328	0.495592	-3.065294
H	3.045536	-0.362769	-3.613565	H	5.130499	0.012955	-2.322980
H	2.248610	0.956013	-4.477601	H	4.887992	1.487696	-3.271221
H	1.615431	-0.685384	-4.603553	H	4.545167	-0.083680	-3.992687
C	-0.191689	0.861072	-3.141098	C	2.107341	1.253228	-3.564984
H	-0.726291	0.166127	-3.799654	H	2.447903	2.265065	-3.814038
H	-0.089139	1.803915	-3.690993	H	1.082239	1.345542	-3.184452
H	-0.842291	1.057138	-2.278383	H	2.052573	0.693893	-4.506384

C	-0.824996	-0.240295	1.178241	C	-1.023369	-0.517829	0.871804
C	-3.115147	-0.125071	2.184496	C	-3.087948	0.117660	2.013345
C	-4.066187	1.072549	2.266177	C	-3.079409	1.544007	2.577283
H	-4.698620	1.019071	3.165145	H	-3.494324	1.581308	3.590184
H	-4.743701	1.122544	1.402732	H	-3.677822	2.227421	1.961934
H	-3.537815	2.035041	2.315558	H	-2.065803	1.964372	2.625219
C	-3.953264	-1.399041	2.344228	C	-4.536928	-0.358996	1.922231
H	-3.325555	-2.302959	2.284752	H	-4.603323	-1.383168	1.527694
H	-4.715969	-1.490029	1.556009	H	-5.136890	0.276337	1.259840
H	-4.472411	-1.426676	3.306608	H	-5.025793	-0.354314	2.902586
C	-2.029309	-0.021520	3.281604	C	-2.230829	-0.816280	2.925403
H	-2.347310	-0.458017	4.230568	H	-2.848486	-1.468840	3.544860
H	-1.805756	1.032545	3.470655	H	-1.620666	-0.218859	3.607432
C	-0.769720	-0.701994	2.685366	C	-1.282428	-1.611916	1.982443
C	-0.841220	-2.219855	2.868926	C	-1.997355	-2.834712	1.409799
H	0.040987	-2.735355	2.456767	H	-1.379172	-3.392788	0.690605
H	-1.730214	-2.646584	2.377999	H	-2.921970	-2.545918	0.874657
H	-0.900362	-2.493844	3.927963	H	-2.288147	-3.540452	2.195007
C	0.497219	-0.183500	3.359011	C	-0.015866	-2.036304	2.711392
H	0.525389	-0.432292	4.425547	H	0.603330	-2.721767	2.101094
H	0.587640	0.912002	3.284926	H	-0.239916	-2.563934	3.645412
H	1.399085	-0.613430	2.904155	H	0.635745	-1.186029	2.967858
C	-2.775771	0.332131	-0.303769	C	-2.792805	0.696647	-0.465314
C	-3.311247	-0.590347	-1.240261	C	-3.652982	0.026057	-1.378671
C	-3.375285	-2.084653	-0.971210	C	-4.075953	-1.420594	-1.183202
H	-2.831003	-2.309217	-0.014798	H	-3.804077	-1.750647	-0.133633
C	-4.831854	-2.530912	-0.794109	C	-5.589530	-1.599149	-1.341307
H	-4.901545	-3.608477	-0.607661	H	-5.901655	-2.610532	-1.055850
H	-5.448798	-2.313779	-1.672206	H	-5.924665	-1.441032	-2.373095
H	-5.295024	-2.021952	0.067232	H	-6.147636	-0.897031	-0.710803
C	-2.684985	-2.873938	-2.090935	C	-3.300555	-2.313755	-2.158361
H	-1.685227	-2.479925	-2.305469	H	-2.203985	-2.186104	-2.001715
H	-3.247652	-2.844531	-3.031136	H	-3.508229	-2.076579	-3.205968
H	-2.570826	-3.929932	-1.821159	H	-3.530445	-3.373151	-2.009553
C	-3.805861	-0.119895	-2.465079	C	-4.096087	0.697154	-2.525329
H	-4.227896	-0.825759	-3.181897	H	-4.760005	0.188322	-3.225861
C	-3.765592	1.235826	-2.784207	C	-3.696612	2.006754	-2.790654
H	-4.151774	1.585207	-3.741117	H	-4.055725	2.517835	-3.683540
C	-3.224334	2.140923	-1.873722	C	-2.828613	2.651598	-1.914491
H	-3.184201	3.200222	-2.132381	H	-2.505293	3.670565	-2.133867
C	-2.732734	1.712898	-0.631182	C	-2.361516	2.013335	-0.753838
C	-2.147599	2.766366	0.299304	C	-1.383139	2.775873	0.121652

H	-1.919669	2.298084	1.291275	H	-1.154229	2.177526	1.046719
C	-3.139439	3.915548	0.557070	C	-1.976450	4.116432	0.574479
H	-3.722369	3.723130	1.498670	H	-2.965643	3.988216	1.031115
H	-3.877458	4.043993	-0.277820	H	-2.096413	4.822227	-0.255439
H	-2.603626	4.893837	0.677632	H	-1.335498	4.602969	1.318897
C	-0.827064	3.283491	-0.278666	C	-0.065641	2.984475	-0.633054
H	-0.119280	2.456996	-0.441233	H	-0.190286	3.586676	-1.539560
H	-0.337379	3.987625	0.408716	H	0.372954	2.026197	-0.939412
H	-0.957520	3.796580	-1.234015	H	0.673473	3.498180	-0.006851
B	-0.073139	-1.341539	0.307145	B	-0.373828	-1.156745	-0.398889
H	-0.374056	0.765156	1.129859	H	-0.354590	0.241607	1.326494
H	0.296034	-1.199466	-1.152268	H	1.171798	-0.743245	-1.745346

References

1. R. Jazzar, R. D. Dewhurst, J.-B. Bourg, B.Donnadieu, Y. Canac and G. Bertrand, *Angew. Chem. Int. Ed.* **2007**, *46*, 2899.
2. L. Brandsma and J. W. Zwikker, *Science of Synthesis* **2005**, *8a*, 243.
3. J. E. Borger, M. S. Bakker, A. W. Ehlers, M. Lutz, J. C. Slootweg and K. Lammertsma, *Chem. Commun.* **2016**, *52*, 3284.
4. K. H. Dötz and W. *J.* Kuhn, *Organomet. Chem.* **1985**, *286*, C23.
5. Sheldrick, G. M. *Acta Crystallogr. C* **2015**, *71*, 3.
6. C. F. Macrae, I. J. Bruno, J. A. Chisholm, P. R. Edgington, P. McCabe, E. Pidcock, L. Rodriguez-Monge, R. Taylor, J. van de Streek and P. A. Wood, *J. Appl. Cryst.* **2008** *41*, 466.
7. Persistence of Vision Raytracer (Version 3.6), Persistence of Vision Pty. Ltd., **2004**.
8. M. J. Frisch, G. W. Trucks, H. B. Schlegel, G. E. Scuseria, M. A. Robb, J. R. Cheeseman, G. Scalmani, V. Barone, B. Mennucci, G. A. Petersson, H. Nakatsuji, M. Caricato, X. Li, H. P. Hratchian, A. F. Izmaylov, J. Bloino, G. Zheng, J. L. Sonnenberg, M. Hada, M. Ehara, K. Toyota, R. Fukuda, J. Hasegawa, M. Ishida, T. Nakajima, Y. Honda, O. Kitao, H. Nakai, T. Vreven, J. A. Montgomery, Jr., J. E. Peralta, F. Ogliaro, M. Bearpark, J. J. Heyd, E. Brothers, K. N. Kudin, V. N. Staroverov, T. Keith, R. Kobayashi, J. Normand, K. Raghavachari, A. Rendell, J. C. Burant, S. S. Iyengar, J. Tomasi, M. Cossi, N. Rega, J. M. Millam, M. Klene, J. E. Knox, J. B. Cross, V. Bakken, C. Adamo, J. Jaramillo, R. Gomperts, R. E. Stratmann, O. Yazyev, A. J. Austin, R. Cammi, C. Pomelli, J. W. Ochterski, R. L. Martin, K. Morokuma, V. G. Zakrzewski, G. A. Voth, P. Salvador, J. J. Dannenberg, S. Dapprich, A. D. Daniels, O. Farkas, J. B. Foresman, J. V. Ortiz, J. Cioslowski and D. J. Fox, Gaussian09 rev. E.01, Gaussian, Inc., Wallingford CT, 2013.
9. a) M. Svensson, S. Humbel, R. D. J. Froese, T. Matsubara, S. Sieber and K. Morokuma, *J. Phys. Chem.* **1996**, *100*, 19357; b) S. Dapprich, I. Komáromi, K. S. Byun, K. Morokuma and M. J. Frisch, *J. Mol. Struct. (Theochem)* **1999**, *462*, 1; c) T. Vreven, K. S. Byun, I. Komáromi, S. Dapprich, J. A. Montgomery Jr., K. Morokuma and M. J. Frisch, *J. Chem. Theory Comput.* **2006**, *2*, 815.
10. Y. Zhao and D. G. Truhlar, *Acc. Chem. Res.* **2008**, *41*, 157.
11. J. J. P. Stewart, *J. Mol. Model.* **2007**, *13*, 1173.
12. A. V. Marenich, C. J. Cramer and D. G. Truhlar, *J. Phys. Chem. B* **2009**, *113*, 6378.
